# In Vitro Effects of Selective COX and LOX Inhibitors and Their Combinations with Antineoplastic Drugs in the Mouse Melanoma Cell Line B16F10

**DOI:** 10.3390/ijms22126498

**Published:** 2021-06-17

**Authors:** Ines Da-Costa-Rocha, Jose M. Prieto

**Affiliations:** 1 The School of Pharmacy, University of London, 29-39 Brunswick Square, London WC1N 1AX, UK; rocha.ines@gmail.com; 2 School of Pharmacy and Biomolecular Sciences, Liverpool John Moores University, Liverpool L3 3AF, UK

**Keywords:** melanoma, cyclooxygenases, lipoxygenases, eicosanoids, cytotoxicity, cell migration, synergy

## Abstract

The constitutive expression or overactivation of cyclooxygenase (COX) and lipoxygenase (LOX) enzymes results in aberrant metabolism of arachidonic acid and poor prognosis in melanoma. Our aim is to compare the in vitro effects of selective COX-1 (acetylsalicylic acid), COX-2 (meloxicam), 5-LOX (MK-886 and AA-861), 12-LOX (baicalein) and 15-LOX (PD-146176) inhibition in terms of proliferation (SRB assay), mitochondrial viability (MTT assay), caspase 3-7 activity (chemiluminescent assay), 2D antimigratory (scratch assay) and synthesis of eicosanoids (EIA) in the B16F10 cell line (single treatments). We also explore their combinatorial pharmacological space with dacarbazine and temozolomide (median effect method). Overall, our results with single treatments show a superior cytotoxic efficacy of selective LOX inhibitors over selective COX inhibitors against B16F10 cells. PD-146176 caused the strongest antiproliferation effect which was accompanied by cell cycle arrest in G_1_ phase and an >50-fold increase in caspases 3/7 activity. When the selected inhibitors are combined with the antineoplastic drugs, only meloxicam provides clear synergy, with LOX inhibitors mostly antagonizing. These apparent contradictions between single and combination treatments, together with some paradoxical effects observed in the biosynthesis of eicosanoids after FLAP inhibition in short term incubations, warrant further mechanistical in vitro and in vivo scrutiny.

## 1. Introduction

Despite the latest advances in adjuvant combination therapeutic strategies most patients with metastatic melanoma still have poor prognosis [1,2]. Therefore, it is important to continue to study newer strategies to increase melanoma patients’ survival.

Epidemiological [3,4], clinical [5,6,7] and animal studies [8,9] have shown that constitutive expression or overactivation of cyclooxygenase (COX) and lipoxygenase (LOX) enzymes during carcinogenesis results in aberrant metabolism of arachidonic acid. The autocrine and paracrine role of prostaglandins and leukotrienes in tumour epithelial cell proliferation, apoptosis, migration and invasion have been reviewed by Wang and DuBois [10]. 

Therefore, the idea of using COX and LOX inhibitors together with other chemotherapy agents is a promising research field in oncology. Synergetic effects of COX inhibitors with several anti-cancer agents have attracted more attention perhaps due to the clinical availability of a variety of NSAIDs vs. the experimental nature of the selective LOX inhibitors. Clinical data on combinatorial therapies involving NSAID and other medication such as anti-PD-1 therapy or sorafenib [11,12] are not compelling, despite preclinical studies suggesting a potentially synergistic relationship. 

**Figure 1:**
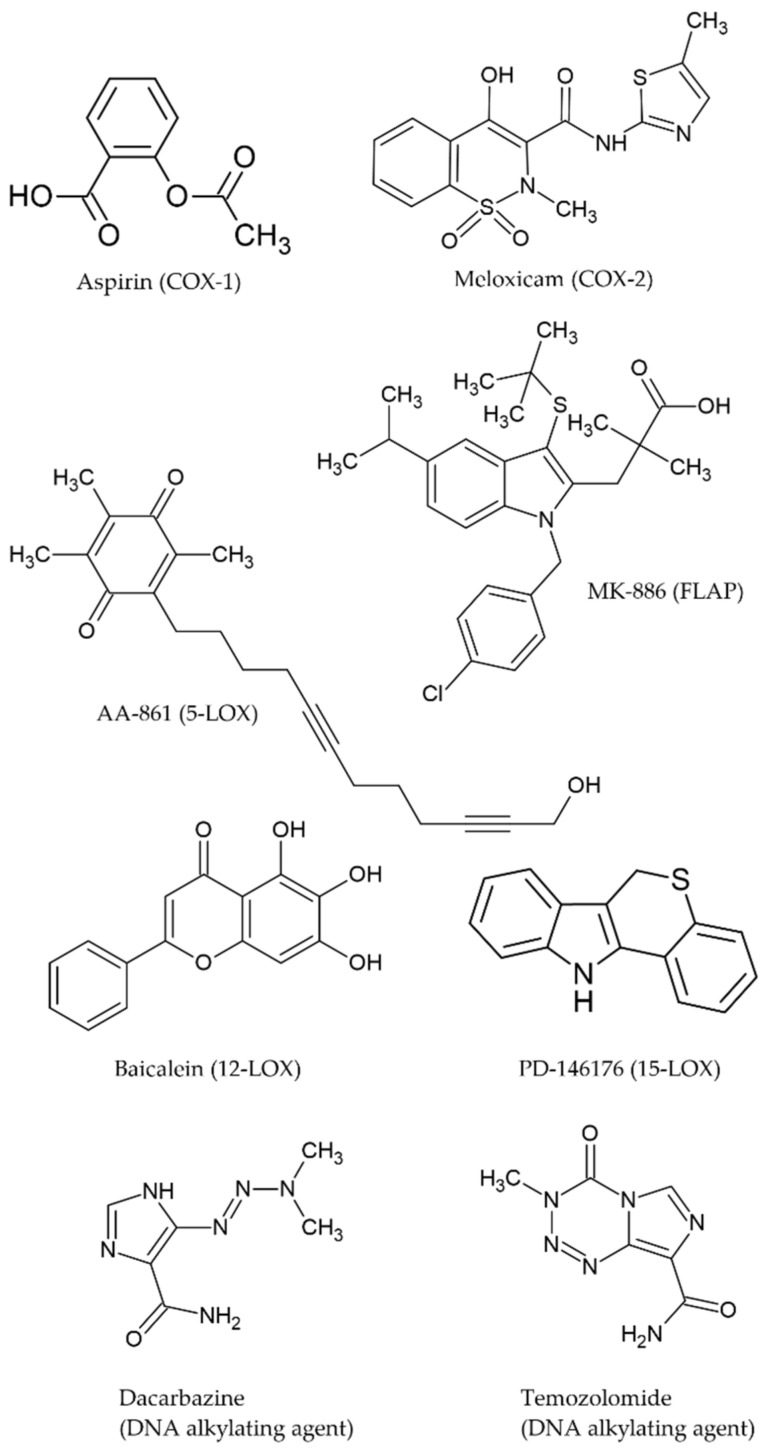
Chemical structures of the selected COX/LOX inhibitors and chemotherapy drugs (from MDL mol files downloaded from ChemSpider and processed with ACDLabs ChemSketch).

More in vitro efforts are needed to reveal new potential synergies between COX/LOX inhibitors and chemotherapy drugs. There have been a few attempts with a variety of cell lines and conditions which do not allow for clear comparisons. Our aim is to compare the effects of a panel of selective COX-1 (acetylsalicylic acid or ASA), COX-2 (meloxicam or MEL), 5-LOX (MK-886 and AA-861), 12-LOX (baicalein) and 15-LOX (PD-146176) inhibitors and their in vitro synergies with two classic antineoplastic drugs used in melanoma treatment: dacarbazine (or DTIC) and temozolomide (or TMZ) (Figure 1) in the same cell line and conditions. 

It is known that temozolomide is a prodrug which spontaneously hydrolyses at physiological pH to yield MTIC, a demethylated form of dacarbazine [13]. Our decision to include two seemingly similar treatments seeks to evidence that this conversion is also effective in the intracellular milieu of the rat B16F10 melanoma cells in vitro, and whether the alkylating effect is better preserved when administered in the form or prodrug.

**Figure 2:**
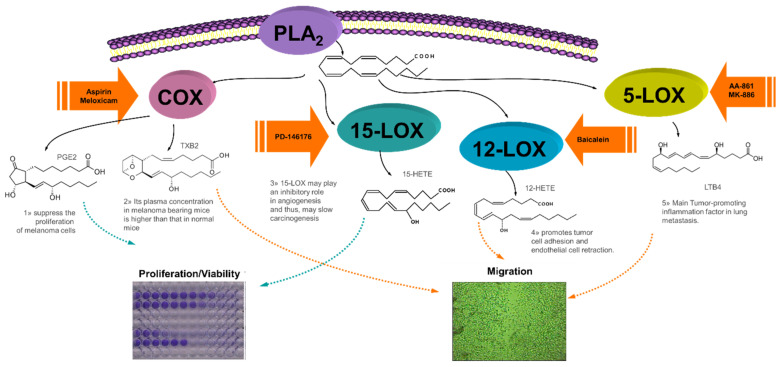
Interplay of COX/LOX metabolites in the proliferation, migration and invasion of melanoma B16F10 cells (authors’ own work).

We selected the well-known B16F10 cell line from *Mus musculus* C57BL/6J strain because is a convenient and widely used experimental model of highly metastatic melanoma to study cytotoxicity, migration, metastatic spread and tissue invasion so it may facilitate other researchers to reproduce or add more data to our results. It is a suitable cell line for our purpose since arachidonic acid (AA) metabolism influences its invasiveness and metastatic activity [14]. Figure 2 shows the interplay of eicosanoids at different stages of the carcinogenesis process in rat melanoma cells B16F10. The mechanism of action of some of the selected inhibitors and B16F10 have been reported for ASA [15,16,17,18,19], MK886 [14], and baicalein [20,21,22,23,24,25]. However, a search in PubMed surprisingly shown almost no data for meloxicam [26], AA-861 or PD-146176 on B16 cells. Of note, there are not reports on the role of 15-LOX on any melanoma cell although some work has been done on other cancer cell lines [27].

## 2. Results

### 2.1. In Vitro Effects of the of the Antineoplastic Drugs Dacarbazine and Temozolomide on B16F10 Cells

**Figure 3:**
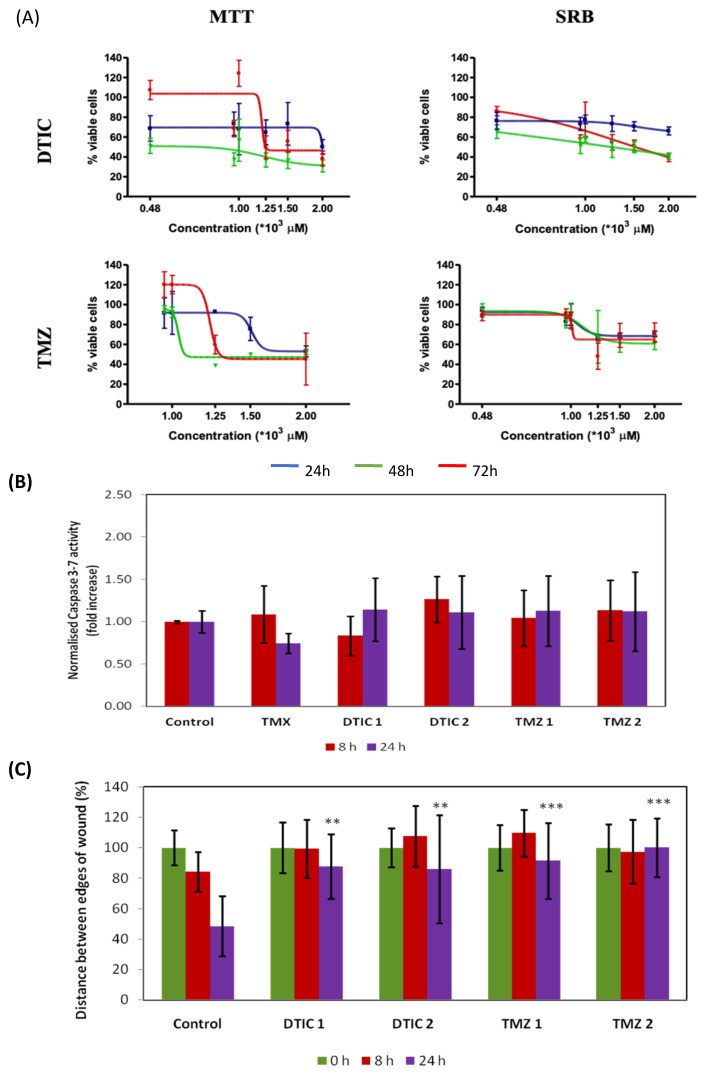
Effects of chemotherapy drugs in B16F10 cells. (**A**) Mitochondrial viability (MTT) and antiproliferative (SRB). (**B**) Caspase 3/7 activity (Y axis shows the increase in Caspase 3-7 activity relative to the control, the luminescent signal was normalised to the number of cells. (**C**) Antimigratory effect in a scratch test. DTIC1 and TMZ1 denote cells incubated with IC_20_. DTIC2 and TMZ2 denote cells incubated with IC_50_. All quantitative values are the mean of three independent assays run in duplicate. Error bars represent SD. Statistical significance by two-tailed unpaired *t*-test with 95% confidence interval (*** *p* < 0.001 ** *p* < 0.01).

The effect of the reference drugs dacarbazine and temozolomide on proliferation, mitochondrial viability, caspases activity and 2D migration in B16F10 melanoma cells was evaluated (Figure 3). The cell cycle profile of B16F10 cells was not influenced by the solvent (DMSO) (Data not shown). Vincristine (VCR) was used as a positive control in this assay, and it caused a G1 phase arrest after 24 h only at 1 nM with 87.78% of the cells being in G1 phase. Sawada et al. [28] reported that the same treatment caused cell cycle arrest in B16F10 cells in G2/M phase after 36 h of incubation. Tamoxifen was also included in our study as a positive control as it is considered a reference apoptosis-inducing agent in human melanoma cell line (A375) (10 µM, 24 h) [29], but it did not have any major effect on the activation of caspase 3/7 in B16F10 under the same conditions (Figure 3B). None of the antineoplastic drugs showed toxic effect at sub-mM concentrations (Figure 3A; see Supplementary Materials for all IC_50_ and IC_20_ values). The IC_50_ determined for both drugs were very similar after 72 h incubation in both assays. Neither DTIC nor TMZ shown any significant effect on activating caspases 3/7 at the concentrations tested (1-2 mM) (Figure 3B). The chemotherapeutic drugs had a significant inhibitory effect on migration when compared to control (Figure 3C). Previous reports on TMZ (10 µM) did not report any activity in the scratch-wound assay [30] but our study shows this is the case at higher concentrations.

### 2.2. In Vitro Effects of the COX Inhibitors Meloxicam and ASA on B16F10 Cells

**Figure 4:**
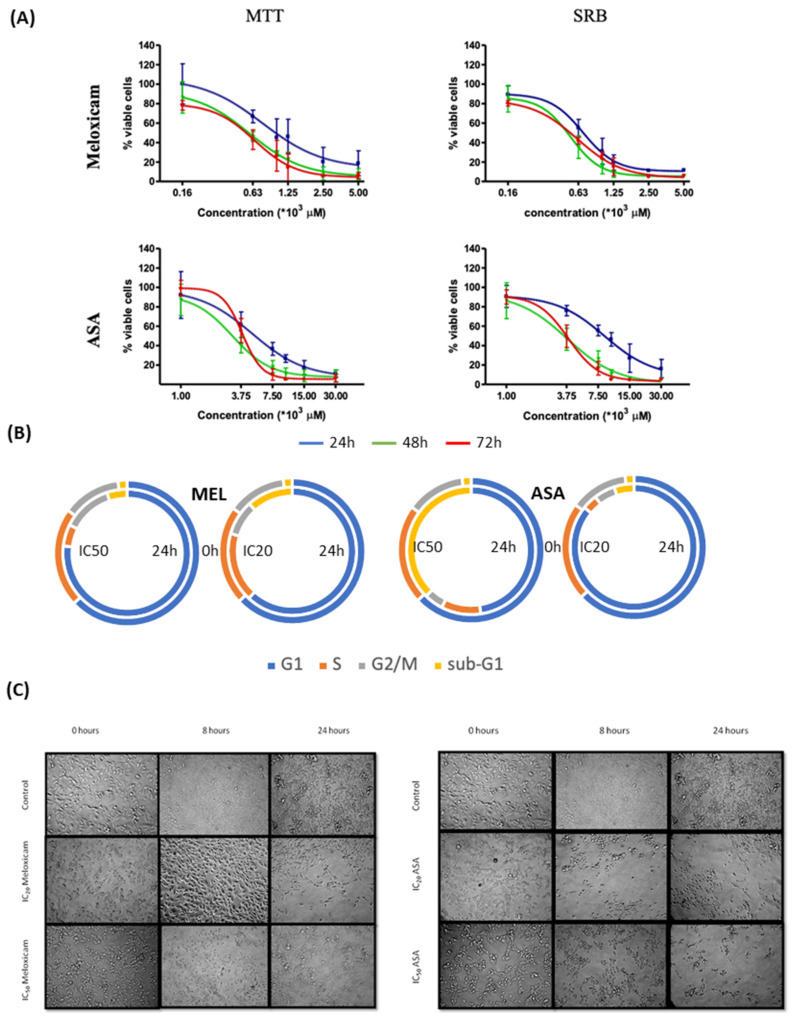
Effects of meloxicam and aspirin (ASA) on proliferation, mitochondrial viability and morphology of B16F10 cells. (**A**) Mitochondrial viability (MTT assay) and antiproliferative (SRB assay). (**B**) Cell cycle distribution (%) after 24 h incubation with IC_20_ and IC_50_ of the inhibitors. (**C**) Cell morphology of treated cells. Values are the mean of three independent assays. Error bars represent SD.

The effects of the COX inhibitors on B16F10 cell viability/proliferation are displayed in Figure 4. All IC_20_ and IC_50_ values were always similar for each drug and in both methods, except for ASA at 24 h (see Supplementary Materials for all IC_50_ and IC_20_ values). In this case a disparity between the mitochondrial viability (measured by the MTT assay) and total biomass (measured by the SRB assay) reflects a differential effect which disappears at longer incubation times. 

The exposure of the cells to meloxicam induced a visible increase of clusters of detached cells. This correlates with cell arrest either at sub-G_1_ (IC_20_ = maximum non-toxic concentration) or G_1_-phase (IC_50_). Treatment with ASA at IC_20_, induced cell arrest in G_1_ phase, a decrease of cells in G_2_/M and an increase in sub-G_1_ phase as from 16 h onwards. The number of cells in S phase also decreased after 24 h. At the IC_50_, ASA causes a great increase of sub-G_1_ population at 24 h. Microcopy evidence extensive cell death at 24 h. However, the effect of the COX inhibitors on caspase activity were not significant in comparison with control (data not shown).

**Figure 5:**
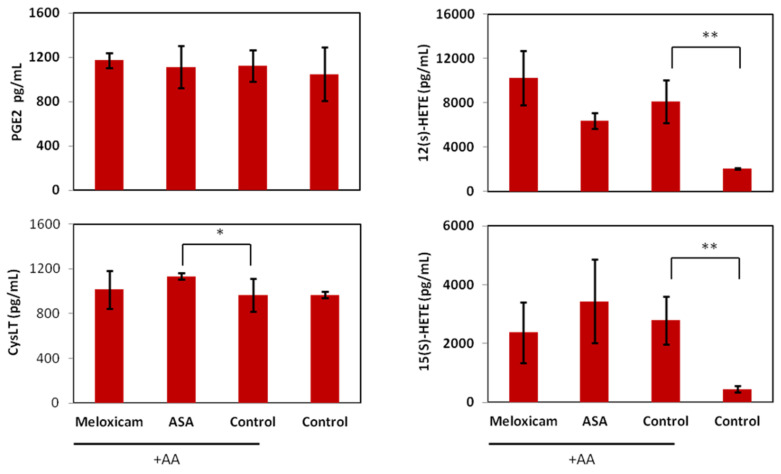
Effects of meloxicam and aspirin (ASA) on eicosanoid biosynthesis. Biosynthesis of PGE2, CysLT, 12(S)- and 15(S)-HETE acid (pg/mL) after 3 h incubation with IC_50_ of the inhibitors plus 10 µM AA. Values are the mean of three independent assays. Error bars represent SD. Statistical significance by two-tailed unpaired *t*-test (** *p* < 0.01 * *p* < 0.05).

Figure 5 shows that stimulation of the cells with exogenous arachidonic acid (control + AA) did not result in any significant increase of PGE_2_ or CysLT synthesis when compared with the control without AA. By contrast, the amount of 12(S)-HETE and 15(S)-HETE synthesised in the presence of exogenous AA was 4- and 6-fold increased with respect to solvent controls. Neither of the COX inhibitors induced a decrease in either PGE_2_, 12(S)- or 15(S)-HETE production. However, the COX-1 inhibitor ASA significantly increased the production of CysLT when compared to control + AA (*p* < 0.05). This may be due to the shunting of excess AA to the 5-LOX pathway, a phenomenon reported in other cancer cells [31]. 

### 2.3. In Vitro Effects of the 5-LOX Inhibitors AA-861 and MK-886 on B16F10 Cells

**Figure 6:**
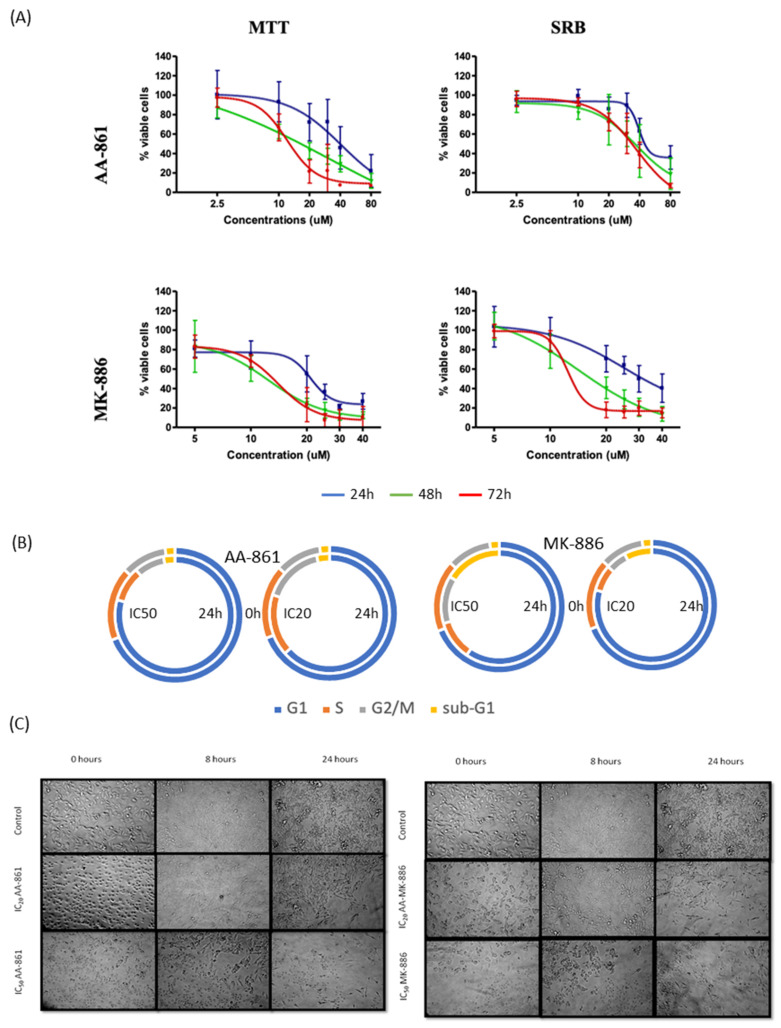
Effects of AA-861 and MK-886 on proliferation, mitochondrial viability and morphology of B16F10 cells. (**A**) Mitochondrial viability (MTT assay) and antiproliferative (SRB assay). (**B**) Cell cycle distribution (%) after 24 h incubation with IC_20_ and IC_50_ of the inhibitors. (**C**) Cell morphology of treated cells. Values are the mean of three independent assays. Error bars represent SD. Statistical significance by two-tailed unpaired *t*-test.

The effects of the LOX inhibitors on B16F10 cell viability/proliferation are displayed in Figure 6. All IC_20_ and IC_50_ values were always similar for each drug and in both methods, except for AA-861 at its non-toxic concentrations (see Supplementary Materials for all IC_50_ and IC_20_ values). In this case there was more effect on proliferation than mitochondrial viability. MK-886 was able to induce cell cycle arrest in G_1_ and a decrease of the number of cells in S and G_2_/M phase even at its IC_20_ whereas AA-861 only affected cell cycle at IC_50_. Addition of AA-861 or MK-886 results in an immediate change of morphology (cells become rounded but do not detach) but the cells recover within a few hours and remain in their normal shape throughout the rest of the experiment. It was observed that MK-886 at IC_50_ was able to induce extensive cell death at 24 h after cell arrest at the sub-G_1_ phase. The caspase 3/7 activity was not affected by any of the 5-LOX inhibitors at either concentration tested in relation to control (data not shown).

**Figure 7:**
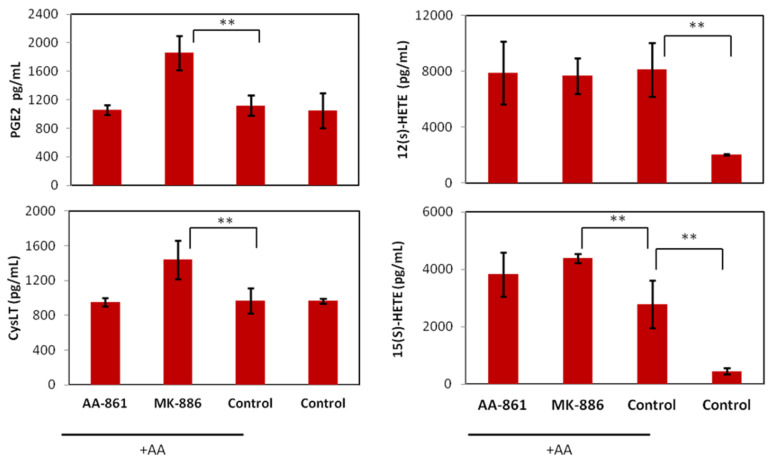
Effects of AA-861 and MK-886 eicosanoid biosynthesis by B16F10 cells. Biosynthesis of PGE2, CysLT, 12(S)- and 15(S)-HETE acid (pg/mL) after 3 h incubation with IC_50_ of the inhibitors plus 10 µM AA. Values are the mean of three independent assays. Error bars represent SD. Statistical significance by two-tailed unpaired *t*-test (** *p* < 0.01).

In terms of eicosanoids production (Figure 7) only MK-866 significantly affected the release of PGE2, CysLT and 12(S)-HETE (*p* < 0.01) after very short incubationsyy8 (3 h). The increase in PGE2 and 12(S)-HETE was expected the in terms of AA shunting but the effect on leukotrienes is paradoxical and cannot be interpreted without further experimentation.

### 2.4. In Vitro Effects of the 12- and 15- Inhibitors Baicalein and PD-146176 on B16F10 Cells

**Figure 8:**
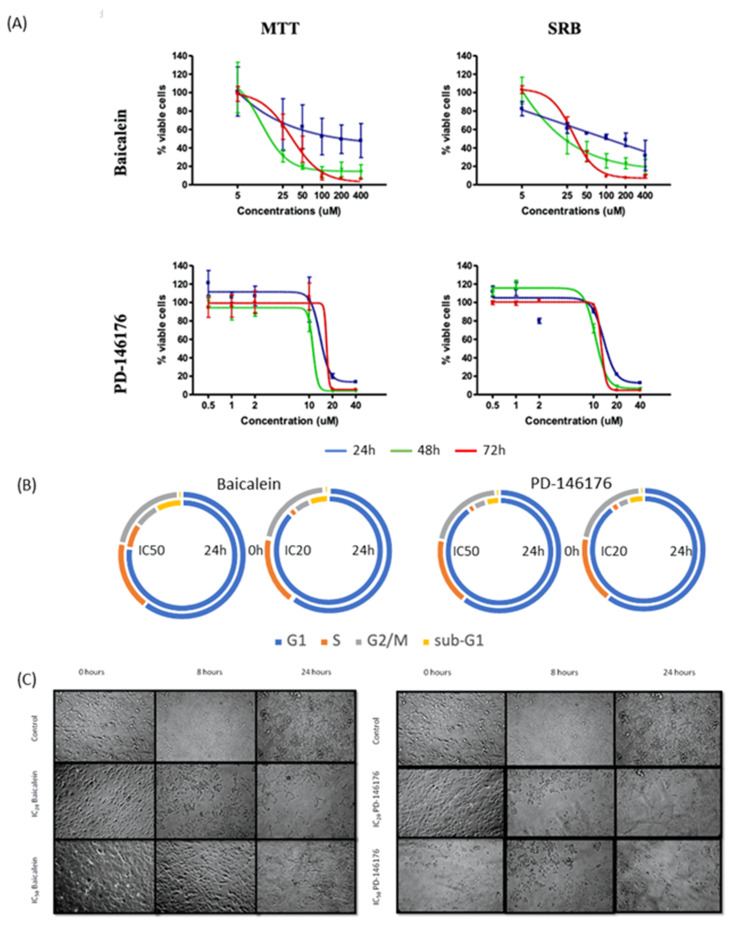
Effects of baicalein and PD-146176 on proliferation, mitochondrial viability, and morphology of B16F10 cells. (**A**) Mitochondrial viability (MTT) and antiproliferative (SRB). (**B**) Cell cycle distribution (%) after 24 h incubation with IC_20_ and IC_50_ of the inhibitors. (**C**) Cell morphology of treated cells. Values are the mean of three independent assays.

The effects of the 12- and 15-LOX inhibitors on B16F10 cell viability/proliferation are displayed in Figure 8. The IC_50_ and IC_20_ values calculated for each drug were similar in both assays except for the IC_50_ of baicalein at 48 h which is three times higher in the SRB assay (see Supplementary Materials for all the IC_50_ and IC_20_ values). Addition of baicalein results in an immediate change of morphology (cells become rounded but do not detach) but the cells recover within few hours and remain in their normal shape throughout the rest of the experiment. Baicalein caused cell arrest in G_1_ phase from 8 h onwards with decreased of the percentage of cells in S and G_2_/M phase while the percentage of cells in sub-G_1_ phase increased. The 15-LOX inhibitor, PD-146176 showed similar effect on the B16F10 cell cycle profile at both concentrations. It induced cell cycle arrest in G_1_ phase at 8 h with decrease of cells in S and G_2_/M phase. The effect on cell morphology is visible observed after 16 h with cells becoming smaller and rounded.

**Figure 9:**
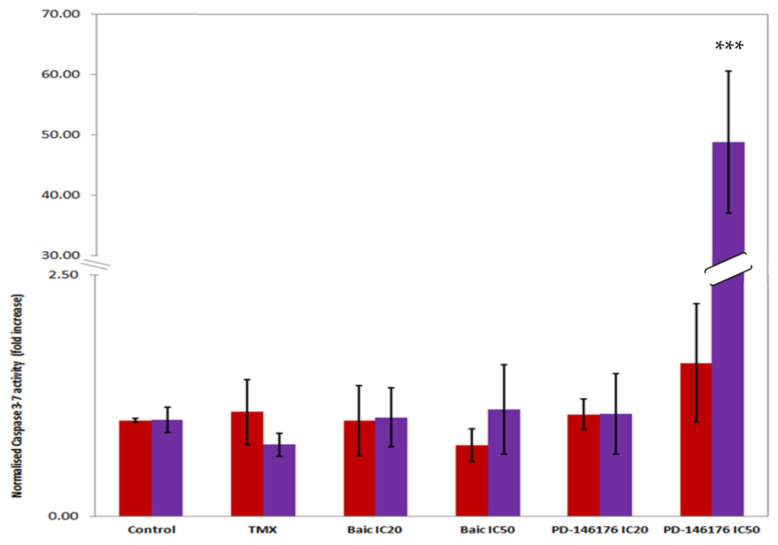
Caspase 3/7 activity in B16F10 incubated with 5-LOX inhibitors at IC_20_ (red bars) and IC_50_ (purple bars) concentrations, tamoxifen at 10 µM (TMX) at 8 and 24 h. The Y axis shows the increase in Caspase 3–7 activity relative to the control. The luminescent signal was normalized to the number of cells (fold increase). The error bars show the standard deviation given from three independent assays. Error bars represent SD. Statistical significance by two-tailed unpaired *t*-test (*** *p* < 0.001).

Baicalein did not have any effect on caspase 3/7 activity but PD-146176 significantly induced its activity at its IC_50_ (Figure 9). Unexpectedly, the treatments with baicalein and PD-146176 did not show any effect on the biosynthesis of any of the eicosanoids even though we were expecting a decrease on the production of 12(S)- and 15(S)-HETE respectively (data not shown).

### 2.5. In Vitro Effect of COX and LOX Inhibitors on Cell 2D Migration/Motility

**Figure 10:**
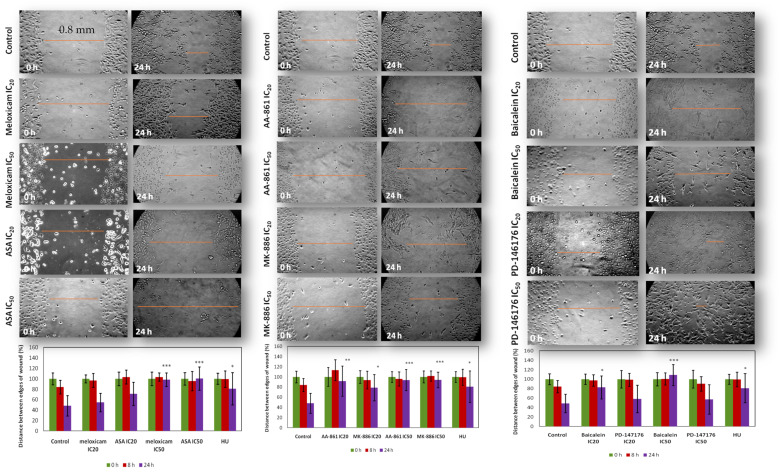
In Vitro effect of COX and LOX inhibitors on B16F10 cell 2D migration/motility. (**Top**) Time lapsed images of the scratch/wound after treatment. In orange, a representative measurement (**Bottom**) Distances between edges of the scratch (in %) of B16F10 cells after treatment (average of five random measurements per replicate). All quantitative values are the mean of three independent assays run in duplicate. Error bars represent SD. Statistical significance by two-tailed unpaired *t*-test with 95% confidence interval (*** *p* < 0.001 ** *p* < 0.01 * *p* < 0.05).

The ‘scratch’ assay permits to study both growth and migration features of a tumour cell population [32]. In our hands it took B16F10 melanoma cells *c.a.* 24 h to recolonise 50% of the mechanical “wound” inflicted on confluent monolayers. The concentrations used in the migration/motility assay were both the toxic (IC_50_) and non-toxic concentrations (IC_20_) determined in the SRB assay at 24 h (Figure 10). Hydroxyurea (HU) is a cell cycle inhibitor–specific for the S phase of cell division [33,34]. It was used as a positive control for proliferation as in this assay proliferation occurs simultaneously with cell migration. This would allow differentiating the contributions of cell proliferation and migration of the wound closure. 

The COX inhibitors were not able to inhibit cell directional migration/motility at toxic concentrations (*p* < 0.01) only. In contrast, the two 5-LOX inhibitors were able to significantly inhibit cell directional migration/motility at both concentrations with AA-861 (*p* < 0.01) having a greater significant effect than MK-886 (*p* < 0.05) at non-toxic concentrations. Both inhibitors had the same significant inhibitory (*p* < 0.001) effect at toxic concentrations though.

### 2.6. Effect of the COX/LOX Inhibitors and Antineoplastic Drugs in Combinatorial Treatments on the Proliferation of B16F10 Cells

**Figure 11:**
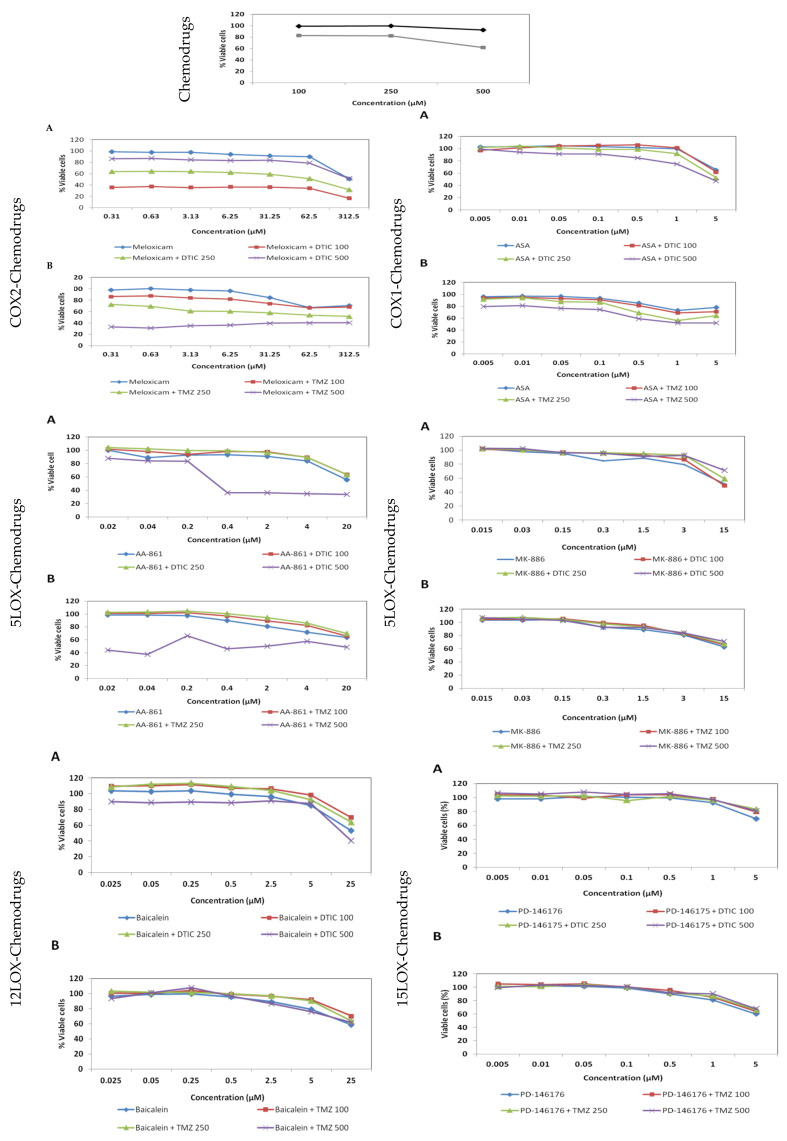
Dose response curves of (**A**) Combination treatment inhibitor + dacarbazine (100, 250 and 500 µM). (**B**) Combination treatment inhibitor + temozolomide (100, 250 and 500 µM). The y axis shows the percentage of viable cells after 72 h and the x axis the different concentrations (µM) of inhibitor.

Figure 11 and Table 1 show that COX inhibitors were more successful than LOX inhibitors in combination treatments: meloxicam led to significant synergies at all concentrations tested, ASA led to mostly neutral or slightly additive effects and the rest only shown antagonisms if at all. Some of the odd strong synergisms such as the one of AA-861 with the highest concentration of DTIC (500 µM) may soon turn as antagonism as soon as the antineoplastic drug starts being eliminated in vivo. The antagonistic effect was stronger in the combination treatment of TMZ and baicalein. PD-145176 antagonised DTIC or TMZ at all concentrations and the dose response curves show that the percentage of viable cells do not diminish when both drugs are taken together. 

## 3. Discussion

### 3.1. Single Treatments with COX/LOX Inhibitors in B16F10 Cells

Collectively, LOX inhibitors were more cytotoxic than COX inhibitors in single treatments, as shown by their IC_50_s in the millimolar and micromolar range, respectively. Thus, LOX inhibitors are a thousand times more potent than the COX inhibitors suggesting that the inhibition of the LOX pathway is more important to stop cell proliferation/viability than the inhibition of COX pathway in B16F10. Of note, DTIC and TMZ displayed weak in vitro cytotoxic activity against melanoma cells and this agrees with a previous study with TMZ in human (C-32, HT-144, and SKMEL-28) and mouse (B16F10) melanoma cell populations [30]. These drugs need of longer timeframes to exert their toxic effects. The order of cytotoxicity for the compounds in terms of IC_50_ was PD-146176 > MK-886 > Baicalein > A-861, similarly to the order of ‘maximum non-toxic concentrations’. This is in line with other studies showing that 5-LOX inhibitors induce cytotoxic and anti-proliferative effects in cultured tumour cells as this has been branded as evidence for an important involvement of 5-LOX in tumourigenesis [10]. Interestingly, 5-LOX inhibitors can reduce the viability of B16F10 and exert cell cycle arrest within 24 h without concurrently activating caspases, so any such pro-apoptotic effect may kick off at later time. Despite this, both selected inhibitors were able to inhibit cell directional migration/motility even at non-toxic concentrations. 

Overall, our results indicate that B16F10 keep a similar baseline eicosanoid production in both the COX and 5-LOX pathways, with 12- and 15-LOX products only reaching similar production of eicosanoids when in presence of excess free AA. Cytotoxic concentrations of the COX and 5-LOX inhibitors do not totally cancel PGE2 or Cys-LT production, and in these cases the AA which is not used by the inhibited pathway is channelled to the other pathway (‘shunting’). In physiological conditions where endogenous AA only is available this shunting may abolish the ‘anticancer’ effect of the treatment. Similar effects were previously reported in colorectal cancer cell [31]. Paradoxically, MK-886 treatment increased the production of CysLT indicating that blocking the 5-lipoxygenase activating protein (FLAP) stimulates the activity of virtually all the arachidonate pathway in B16 cells including 5-LOX. This unexpected result warrants further studies. 

Nonpublished preliminary results indicate no effect in any of the COX or LOX isoform expression by means of treatment with their selective inhibitors [35].

Baicalein suppressed cell proliferation of B16F10 melanoma cells by arresting cells in G_1_ phase as well as cell directional migration. This drug did not show an effect on caspase 3/7 activities at 24 h. Longer incubation times might be necessary to observe a decrease in 12(S)-HETE production. 

The 15-LOX inhibitor PD-146176 was the best single in vitro antiproliferative treatment. It can be suggested that this is because it causes strong cell cycle arrest in G_1_ phase. The drug—at its IC_50—_did not show any inhibitory effect on cell directional migration but greatly increased the activity of the caspases. It can also be speculated that the ‘scratch’ inflicted on the cell monolayer in the migration assay activates mechanisms which protect them against or delays the pro-apoptotic effect of the drug. The importance of the 15-LOX pathway in melanoma development warrants further investigation.

### 3.2. Combinatorial COX/LOX Inhibitors-Antineoplastic Drug Treatments

Overall, the combination treatments between the chemotherapeutic drugs and the COX or LOX inhibitors in the B16F10 cell line seem to afford better results for the COX inhibitors (meloxicam > ASA) than for the LOX inhibitors (AA-861 > baicalein > PD-146176 > MK-886). 

One of the goals of combination therapy is lowering the doses of the combined drugs. In this regard, we were able to reduce the concentration of DTIC to one tenth of its IC_50_ (1000 to 100 µM) and meloxicam more than 2000-fold its IC_50_ (0.61 × 10^−3^ to 0.3 µM) and still have a very strong cytotoxicity (~60% cell death). The combination of DTIC or TMZ with meloxicam produced significant synergistic cell death in the murine melanoma cell line B16F10 at all the concentrations tested. Interestingly, the synergetic effect decreased as the concentration of chemotherapeutic drugs increased. A possible explanation might be the formation of a π-π stack between both drugs at those concentrations, the complex being inactive. Combinations of TMZ with meloxicam showed weaker synergism as compared with DTIC. A possible inhibition of PARP by meloxicam may play, at least in part, a mechanistical role in the synergies with methylating agents like DTIC and TMZ. The main function of PARP—which is present in B16F10 cells [36]-is recognizing DNA breaks and facilitating DNA repair via the polyADP-ribosylation of various DNA binding and repair proteins [37]. A previous study with human KB cells (cell line derived from a human carcinoma of the nasopharynx) treated with 50 µM of celecoxib (a COX-2 inhibitor) showed a 3.3-fold increase on cleaved PARP fragment [38]. The combination of methylating agents with meloxicam may therefore induce DNA damage whilst inhibiting its repair. It was also reported that both aspirin and salicylate induced DNA fragmentation and the proteolytic cleavage of PARP in B-cells chronic lymphocytic leukemia (B-CLL) [39]. A clinical trial with patients with metastatic melanoma reported that the combination treatment of TMZ and celecoxib (COX-2 inhibitor) was safe and potentially effective. Nevertheless, it was suggested that randomised studies were needed to explore the role of celecoxib in combination with chemotherapy or as maintenance treatment in these patients [40]. Based on our findings, meloxicam in combination with chemotherapeutic drugs also resulted in strong cytotoxic effects in synergy, thus further supporting the use of the combination of COX-2 inhibitors and chemotherapeutic drugs for the clinical treatment of melanoma.

The combination of antineoplastic drugs and ASA shown that only high concentrations of both drugs resulted in a synergetic to additive effects, these being more significant with TMZ in term of increased cell death. This suggests a less important clinical role for inhibitors of COX-1 as adjuvant therapy. Regarding combination treatments involving LOX inhibitors, the overall results point towards antagonisms with some odd exceptions that are apparently not plausible in vivo.

It is interesting to see how the two seemingly similar drugs, DTIC and MTIC (the active form of the TMZ prodrug) [13] similarly affect the in vitro viability and proliferation of B16F10 cells when administered alone but differently when in combination with the selective COX/LOX inhibitors. Table 1 allows to see how DTIC is usually extending the range of synergic cytotoxic effects in all combination treatments except for AAS, that synergises with TMZ better. 

## 4. Materials and Methods

### 4.1. Drug Treatments

All the drugs were purchased from Enzo Life Sciences (Exeter, UK) except ASA, DTIC and TMZ which were supplied from Sigma (Dorset, UK). Stock concentrations of each drug were made up in dimethyl sulfoxide (DMSO) (Sigma, Dorset, UK), aliquoted and stored at −20 °C until further use. 

### 4.2. Mammalian Cell Culture 

The cell line used for cytotoxicity studies study was the murine melanoma cell line B16F10 (ATCC CRL-647) [41,42,43,44,45,46] The human prostate cancer cells, PC3 (ATCC^®^ CRL-1435™) was used as a control cell line for 5-LOX expression [47,48]. Both cell lines were sub-cultured in Advance RPMI 1640 Medium (Gibco, Waltham, MA, USA) containing 2000 mg/L D-glucose, non-essential amino acids (NEAA) and 110 mg/L sodium pyruvate. The medium was supplemented with 10% heat-inactivated foetal bovine serum-EU (Gibco), 2 mM of L-glutamine (Gibco) and 1% (*v*/*v*) penicillin-streptomycin antibiotic (10,000 units/mL penicillin and 10,000 µg/mL streptomycin) (Gibco). 

### 4.3. Mitochondrial Viability Assay

The 3-(4,5-dimethylthiazol-2-yl)-2,5-diphenyltetrazolium bromide (MTT) assay was performed based on the method previously described by Mosmann [49]. B16F10 cells were seeded at a density of 1 × 104 cells per well in a 96-well tissue culture plate (Nunc, Waltham, MA, USA) and left to adhere overnight. On the following day different concentrations of inhibitors and chemotherapeutic drugs were tested by supplementing them to the growth medium. All the samples (inhibitors, chemotherapeutic drugs, and control (DMSO) were prepared prior to the assay. The cells were incubated with the drugs for 24, 48 and 72 h (5% CO2, 95% O2, 37 °C). At the end of each time point, the media was aspirated, the wells washed with 100 μL of warm PBS and 100 μL of the 0.5 mg/mL MTT solution reagent (0.5 mg/mL) added to each well and the plates incubated for 3 h at 37 °C with 5% CO2. After the incubation, the MTT solution was aspirated and 100 μL of solubilising solution (10% of DMSO in isopropanol (Fisher Scientific, Waltham, MA USA) was added. The optical density (OD) at 570 nm was measured using a Synergy ™ HT multi-detection microplate reader (BioTek Instruments, Inc., Waltham, MA, USA). The percentage of change in cell growth could then be calculated (OD (sample)/OD (blank) × 100). This assay was done using three independent assays performed in triplicate.

### 4.4. Proliferation Assays

The sulphorhodamine B (SRB) assay was used to determine cell density based on the total cellular protein content (biomass by total protein) by staining cellular proteins with SRB [50,51]. As previously described for the MTT assay, 1 × 10^4^ cells per well were seeded in each well of a 96-well tissue culture plate and left overnight in the incubator. The cells were incubated with the compounds for 24, 48, or 72 h similar to the MTT assay. 

At each time point, the cells were fixed with 100 μL cold 40% (*w*/*v*) trichloroacetic acid (Sigma) solution in deionised water. The plates were kept for 1 h at 4 °C and washed five times with water. A SRB solution (0.4% SRB in 0.1% acetic acid) was prepared beforehand. The SRB solution (100 μL per well) was added, and the plates were left at room temperature for 1 h. Subsequently, the plates were rinsed with 1% acetic acid solution, flicked to remove unbound dye, and left to air-dry overnight prior to adding 100 μL of 10 mM TRIS base solution (Sigma) to each well. The plates were left on a shaker for 30 min and the optical density (OD) at 492 nm was measured in a Synergy ™ HT multi-detection microplate reader (BioTek Instruments, Inc.). The percentage change in cell growth was then calculated (OD (sample)/OD (blank) × 100). This assay was performed in triplicate.

### 4.5. Morphology Studies

Cells were observed with an inverted microscope (TMS, Nikon, Amsterdam, Netherlands). Healthy B16F10 cells attach to the bottom of the culture plates and present a mixture of spindle-shaped and epithelial-like morphology. The presence of detached and/or rounded cells, alone or in clusters is evidence for a certain degree of toxic effect. Cell debris indicates destruction of the membrane integrity.

### 4.6. Quantification of Eicosanoids Using Enzyme Immune Assay

Cells were seeded in 24 well tissue culture plate (2 × 10^5^ cells per well in 500 µL growth media) and left overnight to adhere. The cells were treated with the COX or LOX inhibitors for 2 h and subsequently stimulated with arachidonic acid (AA) (5 µL of 10 µM AA) for 1 h. Arachidonic acid solution was previously prepared using 10 mg of AA from porcine liver (99%, from sigma) dissolved in DMSO (final concentration of 30.6 mM). The supernatant was afterwards collected and kept at −80 °C until further use. Different eicosanoids -prostaglandin E_2_ (PGE_2_), cysteinyl leukotrienes, 12-HETE and 15-HETE- were quantified using a specific competitive eicosanoid EIA kit (Enzo, Life Sciences, Exeter, United Kingdom) for each one of them. Each competitive EIA assay was performed according to the manufacturer’s instructions. The samples (in media) were used directly in the EIA kit without any further extraction. Standards were resuspended in media for the calibration curve. Samples for 12- and 15-LOX quantification were prepared in media without FBS due to interference of the FBS with the kit and dilutions of the samples were considered to fit the calibration curve. After different incubations times according to the specific kit, the optical density (OD) was measured at 405 nm and 570 nm using a Synergy ™ HT multi-detection microplate reader (BioTek Instruments, Harwell, United Kingdom). The results obtained are from three independent assays each of which has at least two copies for the same treatment. The quantification of each eicosanoid tested was calculated based on the Percentage of Bound= Net OD/Net B_0_ OD × 100 and the respective calibration curve.

### 4.7. Fluorescence Activated Cell Sorting (FACS) Analysis by Propidium Iodide (PI) Staining

The cells were seeded in 6-well plates (1 × 10^6^ cells per well) overnight. On the following day cells were subjected to the COX and LOX inhibition treatments in 2 mL of growth medium. The cells were harvested at 0, 4, 8, 24 and 48 h after incubation with the inhibitors. Vincristine sulphate (Sigma, Dorset, UK) at 1 nM (in DMSO) for 24 h was used as a positive control in this assay.

The supernatant was collected and the cells trypsinised. The growth medium was removed after centrifugation and the cells washed with PBS (5 mL). The PBS was discarded, and the cells fixed in 1 mL ice-cold 70% ethanol (fisher scientific). This solution was added drop wise to the cell pellet while vortexing to minimise clumping and ensure adequate fixation of all cells. The cells were placed on ice for at least 30 min. Then, the alcohol was spun off and the supernatant aspirated carefully in order not to disturb the cell pellet. The pellet was washed twice with PBS. To ensure that only DNA was stained, the cells were treated with Ribonuclease A (RNase A) solution. The RNase A from bovine pancreas for molecular biology (Sigma) was previously dissolved in PBS (10 mg/mL). This solution was further diluted (1 mg/mL) in PBS buffer and aliquoted into 1.5 mL microfuge tubes and stored at −20 °C until further use. At the time of the experiment, one aliquot of RNase A was dissolved in 9 mL of PBS to give a final concentration of 100 µg/mL and 50 µL were added to each sample. The samples were incubated at room temperature for 15 min. PI solution (450 µL per 1 million cells) at a concentration of 50 µg/mL (stored at −20 °C until further use) was added to each sample. The samples were transferred to ice and kept covered until the flow cytometry analysis was performed (MACSQuant^®^ analyser, Miltenyi Biotec GmbH, Bergisch Gladbach, Germany). A control of unstained cells was first used to set up the parameters for this cells line. The PI signal (20,000 single events) was acquired on a linear scale. A dot plot showing the area under the fluorescence signal versus the height of the PI signal was used to gate out doublets and clumps, which appear outside the linear range of single events. The acquired data was then analysed using MACSQuantify™ software. This assay was performed in triplicate.

### 4.8. Apoptosis (Caspase 3/7 Activity)

Caspase assays were performed using the Caspase-Glo^®^ 3/7 assay kit (Promega, Madison, WI, United States) according to the manufacturer’s protocol. Cells were cultured as described previously in white 96 well plates (Greiner Bio-One Ltd., Kremsmünster, Austria) at 1000 cells per well and left overnight for the cells to bind to the surface. On the following day, the cells were treated with the appropriate regimes of drugs for 8 and 24 h. Tamoxifen (50 µM in ethanol) was used as a positive control in this assay. Afterwards, all the content of the wells with the medium/compound mix was removed and 25 µL of growth medium were added. To each of the wells 25 µL of Caspase-Glo^®^ assay were added and plates were covered with foil and were gently mixed for 60 min at RT. The plates were loaded into the plate reader (FLUOstar optima, BMG, Labtech Ltd., Heathfield, United Kingdom) and the luminescence was measured. To ensure constant conditions the plate reader chamber was maintained at 27 °C throughout the experiment. The experiment was performed in triplicate.

### 4.9. Migration Assay in a 2D Environment (Scratch/Wound Healing Assay) 

The migration assay, or also known as scratch/wound healing assay is an easy, low-cost method to measure cell migration in vitro [52,53]. In this work, the migration assay was performed as follows: cells were seeded and grown to confluence in 24-well tissue culture plate (Nunc). After the monolayer had been formed, a scratch (~5 × 10^5^ cells per well) was created by firmly dragging a 1mL pipette tip through the monolayer. Detached cells were removed by gently washing the well with PBS and the remaining monolayer was treated with the respective inhibitors in 500 μL of growth medium. Hydroxyurea (Sigma) at 0.3 mM in PBS was used as an anti-proliferative agent in this assay (positive control). 

Plates were then placed into the incubator and cell migration was monitored using an inverted microscope (CKx41, Olympus, Southend-on-Sea, United Kingdom) attached to a Nikon Coolpix 4500 digital camera (using 10× magnification). Pictures were taken from 3 independent wells for each treatment at 0, 8 and 24 h. The free Image J 1.43 software (National Institutes of Health, Bethesda, MD, USA) was used to measure the distances between the edges of the scratch in each of the pictures. For each time point, three pictures were taken for each well and five random distances between each gap were then measured in each photo using Image J software to generate an average gap distance value. The migratory capacity was then measured by observing the closing of the gap, and thus the decrease in distance between the edges. The assay was performed in triplicate.

### 4.10. Synergies and Antagonism of Inhibitors and Chemotherapy Drug Combinatorial Treatments

The method used in this paper is the median-effect plot based on the multiple drug-effect equation of Chou-Talalay [54] derived from enzymatic models. When the dose effect relationships of both drugs in single treatments and in combination are all parallel in the median-effect plot this correspond to compounds presenting similar mechanism of actions. Thus, for mutually exclusive compounds (drugs that have the same or similar modes of action):

(*f*_*a*_/*f*_*u*_)_1_^1/*m*^ + (*f*_*a*_/*f*_*u*_)_2_^1/*m*^ = (*D*)_1_/(*D*_*m*_)_1_ + (*D*)_2_/(*D*_*m*_)_2_ (1)

where (*D*)_1_: dose of drug 1, (*D*)_2_: dose of drug 2, (*D*_*m*_)_1_: the median-effect dose of drug 1 and (*D*_*m*_)_2_: the median-effect dose of drug 2.

When the plots of both drugs as single treatments are parallel, but the plot of their combination is concave upwards and can intercept the plot of the more active compound, they have different mechanism of action. For mutual non-exclusive compounds:

(*f*_*a*_/*f*_*u*_)_1_ ^1/*m*^ + (*f*_*a*_/*f*_*u*_)_2_ ^1/*m*^ = (*D*)_1_/(*D*_*m*_)_1_ + (*D*)_2_/(*D*_*m*_)_2_ + (*D*)_1_(*D*)_2_/(*D*_*m*_)_1_(*D*_*m*_)_2_ (2)

The combination index equation and the term combination index (*CI*) were derived from the multiple drug-effect equation [54] and it is basically a quantitative approach to calculate the quality of a drug combination that assign *CI* = 1 for an additive effect, *CI* < 1 for synergism and *CI* > 1 for antagonistic effect. For mutually exclusive compounds:

*CI* = (*D*)_1_/(*D*_*x*_)_1_ + (*D*)_2_/(*D*_*x*_)_2_ = (*D*)_1_/*D*_*m*_(*f*_*a*_/1 − *f*_*a*_))^1/*m*1^ + (*D*)_2_/*D*_*m*_(*f*_*a*_/1 − *f*_*a*_))^1/*m*2^ (3)

For mutually non-exclusive compounds:

*CI* = (*D*)_1_/(*D*_*x*_)_1_ + (*D*)_2_/(*D*_*x*_)_2_ + (*D*)_1_(*D*)_2_/(*D*_*x*_)_1_(*D*_*x*_)_2_ (4)

(*D*)_1_, (*D*)_2_ in the numerators are the doses for the drugs in combination producing x% effect in the experiment. (*D_x_*)_1_, (*D_x_*)_2_ in the denominators are the doses of the drugs alone producing x% effect.

Chou and Talalay define three steps to determine the combination index [54]. First, calculate the median-effect plot and determine m and *D_m_* values for the two drugs and the combinations. Second, for a particular *f*_*a*_ calculate the corresponding dose for each drug and the combination:

*D*_*x*_ = *D*_*m*_ (*f*_*a*_/(1 − *f*_*a*_))^1/*m*^ (5)

To calculate the combination index using the above combination index equations we used Calcusyn©, a software available from Biosoft (Cambridge, UK). Table 2 shows the experimental design of our combination studies.

The outcome of combination treatments was assessed using the SRB assay (1 × 10^4^ cell per well in a 96-well tissue culture plate). Cells were treated with varying ratios of COX and LOX inhibitors and chemotherapeutic drugs (see Table 2) for 72 h. To cover all the range of IC_50_ and IC_20_ concentrations for the COX and LOX inhibitors, the starting values of these drugs for the combination studies were the IC_50_s determined in the SRB assay at 24 h. For the chemotherapeutic drugs, the initial concentration was the IC_50_ determined at 72 h. Because all the combinations were in a non-constant ratio, neither the classic nor the conservative isobolograms could be constructed. This does not affect the significance of the result of the calculations. 

### 4.11. Statistics

Inhibitory concentrations 50% (IC_50_) were calculated from the equation of the best non sigmoidal curve fitting the data points with the help of GraphPad Prism v. 5 (GraphPad Software Inc., La Jolla, CA, USA). Inhibitory concentrations 20 (I) were taken from the minimum experimental concentration exerting about 20% cell death. Comparison of each treatment with the control group was performed with GraphPad Prism v. 5 using the Student’s *t* test. When all treatments were compared to each other, then a one-way Analysis of Variance (ANOVA) was run prior to the Multiple comparison test. Other basic statistical calculations (averages, etc.) were performed with the help of Excel (Microsoft, Redmond, WA, USA).

## 5. Conclusions

The overall results show a superior in vitro cytotoxic efficacy of selective LOX inhibition over selective COX inhibition against B16F10 cells. The 15-LOX inhibition by PD-146176 caused the strongest antiproliferation effect which was accompanied by cell cycle arrest in G_1_ phase and increase of caspases activity. This situation is unexpectedly reversed when the inhibitors are combined with the antineoplastic drugs DTIC or TMZ, with meloxicam/DTIC being the most favorable in theory. These apparent contradictions, together with paradoxical effects in biosynthesis of eicosanoids after FLAP inhibition in short term incubations, may warrant further mechanistical in vitro and in vivo scrutiny.

## Figures and Tables

**Table 1:** Heat map of the synergy-antagonism of combination treatments (cytotoxicity on B16F10 after 72 h incubations). (5S) Very strong synergism (CI < 0.1); (4S) Strong synergism (CI = 0.1–0.3); (3S) Synergism (CI = 0.3–0.7); (2S) Moderate synergism (CI = 0.7–0.85); (1S) Slight synergism (CI = 0.85-0.90); (+) Nearly additive (CI = 0.90–1.1); (1A) Slight antagonism (CI = 1.10–1.20); (2A) Moderate antagonism (CI = 1.20–1.45); (3A) Antagonism (CI = 1.45–3.3); (4A) Strong antagonism (CI = 3.3–10); (5A) Very strong antagonism (CI > 10). Empty cells denote “No combinatorial effect”. All concentrations are in µM.

DTIC				ASA				TMZ				ASA			
	5	10	50	100	500	1000	5000		5	10	50	100	500	1000	5000
100							+	100	3S	2S	+	1S	3S	4S	+
250					2A	3S	+	250	1S	2A	2S	2S	3S	3S	2S
500					3S	3S	+	500	+	+	+	+	3S	3S	3S
			
**DTIC**	**Meloxicam**							**TMZ**	**Meloxicam**						
	0.31	0.63	3.13	6.25	31.25	62.50	312.50		0.31	0.63	3.13	6.25	31.25	62.50	312.50
100	5S	5S	5S	5S	5S	5S	5S	100	4S	4S	4S	3S	3S	3S	1A
250	4S	4S	4S	4S	4S	4S	4S	250	3S	3S	3S	3S	3S	3S	2A
500	3S	3S	3S	3S	3S	3S	3S	500	3S	3S	3S	3S	3S	3S	1S
			
**DTIC**	**AA-861**							**TMZ**	**AA-861**						
	0.02	0.04	0.20	0.40	2.00	4.00	20.00		0.02	0.04	0.20	0.40	2.00	4.00	20.00
100		3A	2S	5A	5A	4A	2S	100				4A	3A	3A	3A
250		5A	5A	5A	5A	4S	+	250					4A	3A	3A
500	3S	3S	3S	5S	5S	5S	4S	500	3S	3S	2S	3S	3S	1S	2A
			
**DTIC**	**MK-886**							**TMZ**	**MK-886**						
	0.015	0.030	0.150	0.300	1.500	3.000	15.000		0.015	0.030	0.150	0.300	1.500	3.000	15.000
100			4A	3A	4A	4A	3S	100				5A	5A	3A	2A
250			3A	5A	5A	5A	2A	250				5A	4A	3A	3A
500			3A	4A	4A	5A	3A	500				3A	4A	3A	3A
			
**DTIC**	**Baicalein**							**TMZ**	**Baicalein**						
	0.025	0.050	0.250	0.500	2.500	5.000	25.000		0.025	0.050	0.250	0.500	2.500	5.000	25.000
100						4A	3A	100				5A	4A	4A	2A
250						3A	3A	250				5A	5A	3A	1A
500	3S	3S	3S	3S	+	2S	+	500				4A	3A	2A	2A
			
**DTIC**	**PD-146176**							**TMZ**	**PD-146176**						
	0.005	0.010	0.050	0.100	0.500	1.000	5.000		0.005	0.010	0.050	0.100	0.500	1.000	5.000
100						2A	2A	100					3A	2A	3A
250						2A	3A	250					3A	3A	3A
500						3A	3A	500					3A	3A	3A

**Table 2:** Experimental design of the combination studies. [CHEMODRUG] refers to the concentrations of chemotherapeutic drugs (DTIC and TMZ) and [DRUG] refers to all the COX and LOX concentrations tested in combination. IC_50_ values for the drugs were the ones obtained in the SRB assay at 24 h and IC_50_ values for the chemotherapeutic drugs were the ones obtained at 72 h.

			[CHEMODRUG]		
		Vehicle	1/10 IC_50_	1/4 IC_50_	1/2 IC_50_
	**Vehicle**	Control	Single_Chemodrug_	Single_Chemodrug_	Single_Chemodrug_
**[DRUG]**	**1/2000 IC_50_**	Single_Drug_	Comb_Drug-Chemodrug_	Comb_Drug-Chemodrug_	Comb_Drug-Chemodrug_
	**1/1000 IC_50_**	Single_Drug_	Comb_Drug-Chemodrug_	Comb_Drug-Chemodrug_	Comb_Drug-Chemodrug_
	**1/200 IC_50_**	Single_Drug_	Comb_Drug-Chemodrug_	Comb_Drug-Chemodrug_	Comb_Drug-Chemodrug_
	**1/100 IC_50_**	Single_Drug_	Comb_Drug-Chemodrug_	Comb_Drug-Chemodrug_	Comb_Drug-Chemodrug_
	**1/20 IC_50_**	Single_Drug_	Comb_Drug-Chemodrug_	Comb_Drug-Chemodrug_	Comb_Drug-Chemodrug_
	**1/10 IC_50_**	Single_Drug_	Comb_Drug-Chemodrug_	Comb_Drug-Chemodrug_	Comb_Drug-Chemodrug_
	**1/2 IC_50_**	Single_Drug_	Comb_Drug-Chemodrug_	Comb_Drug-Chemodrug_	Comb_Drug-Chemodrug_

## Data Availability

Data are available by request to the correspondent author.

## Supplementary Materials

The following are available online at https://www.mdpi.com/article/10.3390/ijms22126498/s1.
