# Supplementary material for: In Vitro Effects of Selective COX and LOX Inhibitors and Their Combinations with Antineoplastic Drugs in the Mouse Melanoma Cell Line B16F10
Source: Int J Mol Sci. 2021 Jun 17;22(12):6498. doi: 10.3390/ijms22126498 (PMC8234702; doi:10.3390/ijms22126498)
Supplement: Supplementary file 1 [file ijms-22-06498-s001.zip › ijms-1216147-supplementary.pdf]

Supplementary Materials

In Vitro Effects of Selective COX and LOX Inhibitors and Their Combinations with Antineoplastic Drugs in the Mouse Melanoma Cell Line B16F10

Ines Da-Costa-Rocha [1] and Jose M. Prieto [1,2,*]

[1] The School of Pharmacy, University of London, 29-39 Brunswick Square, London WC1N 1AX, UK; rocha.ines@gmail.com
[2] School of Pharmacy and Biomolecular Sciences, Liverpool John Moores University, Liverpool L3 3AF, UK
* Correspondence: j.m.prietogarcia@ljmu.ac.uk

Citation: Da-Costa-Rocha, I.; Prieto, J.M. In Vitro Effects of Selective COX and LOX Inhibitors and Their Combinations with Antineoplastic Drugs in the Mouse Melanoma Cell Line B16F10. *Int. J. Mol. Sci.* **2021**, *22*, 6498. https://doi.org/10.3390/ijms22126498

Academic Editor: Jochen Utikal

Received: 25 April 2021
Accepted: 31 May 2021
Published: 17 June 2021

Publisher's Note: MDPI stays neutral with regard to jurisdictional claims in published maps and institutional affiliations.

Copyright: © 2021 by the authors. Licensee MDPI, Basel, Switzerland. This article is an open access article distributed under the terms and conditions of the Creative Commons Attribution (CC BY) license (http://creativecommons.org/licenses/by/4.0/).

1. Cytotoxicity studies

Table S1. Tabulated data for IC50 and IC20 values of single treatments.

		DTIC		TMZ	
		MTT	SRB	MTT	SRB
**24 hours**	IC_{50}	-	-	-	-
**48 hours**	IC_{50}	$1.23*10^3$	$1.27*10^3$	$1.03*10^3$	-
**72 hours**	IC_{50}	$1.21*10^3$	$1.33*10^3$	$1.23*10^3$	$1.01*10^3$

		Meloxicam		ASA	
		MTT	SRB	MTT	SRB
**24 hours**	IC_{20}	$0.16*10^3$	$0.16*10^3$	$1.00*10^3$	$1.00*10^3$
	IC_{50}	$0.76*10^3$	$0.68*10^3$	$4.78*10^3$	$8.64*10^3$
**48 hours**	IC_{20}	$0.16*10^3$	$0.16*10^3$	$1.00*10^3$	$1.00*10^3$
	IC_{50}	$0.59*10^3$	$0.54*10^3$	$3.10*10^3$	$3.80*10^3$
**72 hours**	IC_{20}	$0.16*10^3$	$0.16*10^3$	$1.00*10^3$	$1.00*10^3$
	IC_{50}	$0.65*10^3$	$0.61*10^3$	$3.86*10^3$	$3.89*10^3$

		AA-861		MK-886	
		MTT	SRB	MTT	SRB
**24 hours**	IC_{20}	10.00	20.00	5-10.00	10.00
	IC_{50}	41.03	39.45	20.98	24.99
**48 hours**	IC_{20}	2.50	10.00	<5.00	5-10.00
	IC_{50}	30.50	37.33	12.41	14.34
**72 hours**	IC_{20}	2.50	10.00	5.00	5-10.00
	IC_{50}	12.08	37.61	13.96	12.42

		Baicalein		PD-146176	
		MTT	SRB	MTT	SRB
**24 hours**	IC_{20}	5.00	5.00	1.00	1.00
	IC_{50}	30.98	41.06	13.89	13.64
**48 hours**	IC_{20}	5.00	5.00	1.00	1.00
	IC_{50}	11.43	34.14	11.24	10.69
**72 hours**	IC_{20}	5.00	5.00	1.00	1.00
	IC_{50}	33.68	30.53	16.85	12.65

2. Cell cycle profiles for single drug treatments

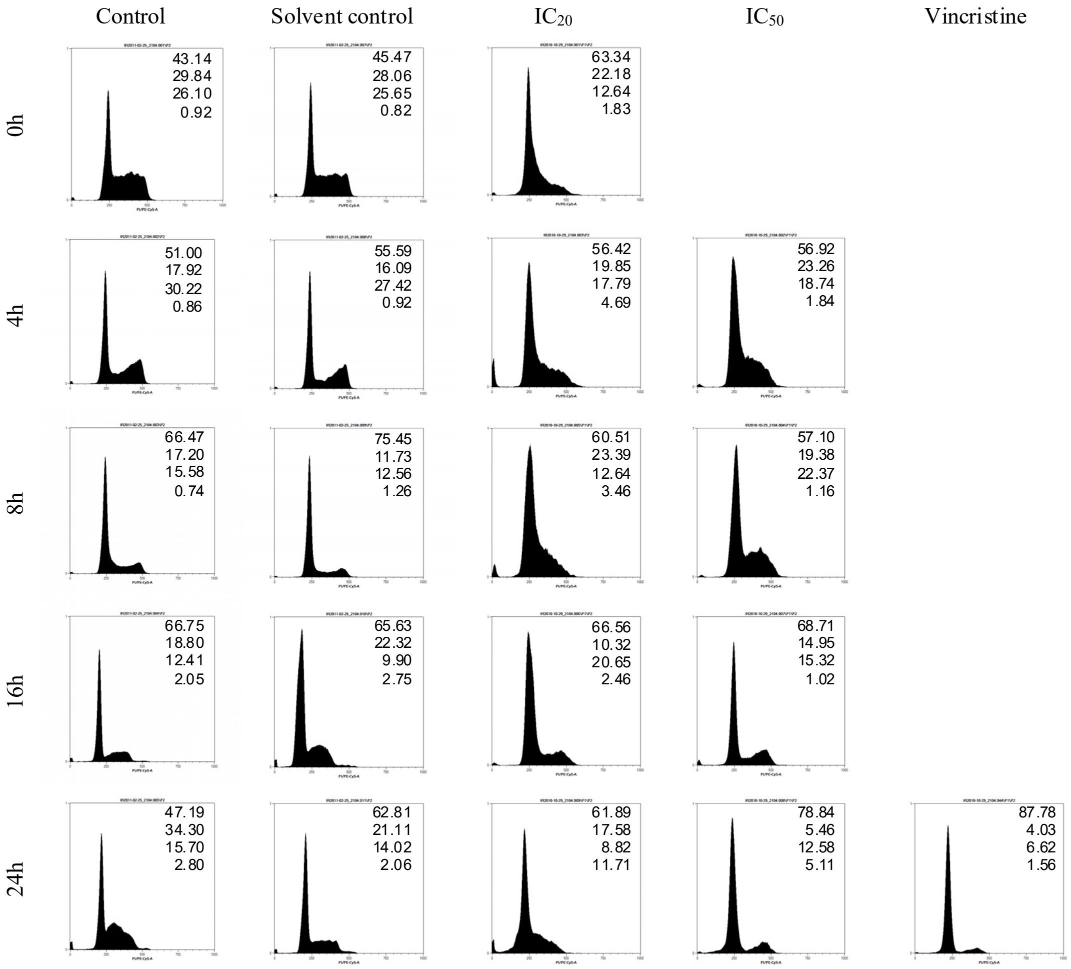

Figure S1. Effects of COX2 inhibitor on cell cycle status. B16F10 cells were treated with IC_{20} and IC_{50} concentration of Meloxicam inhibitor for 0, 4, 8, 16 and 24 hours. Flow cytometric analysis of DNA content was performed after propidium iodide staining. Percentage of cells in each phase of the cell cycle (sub-G_1, G_1, S, and G_2/M) is indicated. Representative data from three independent experiments are shown.

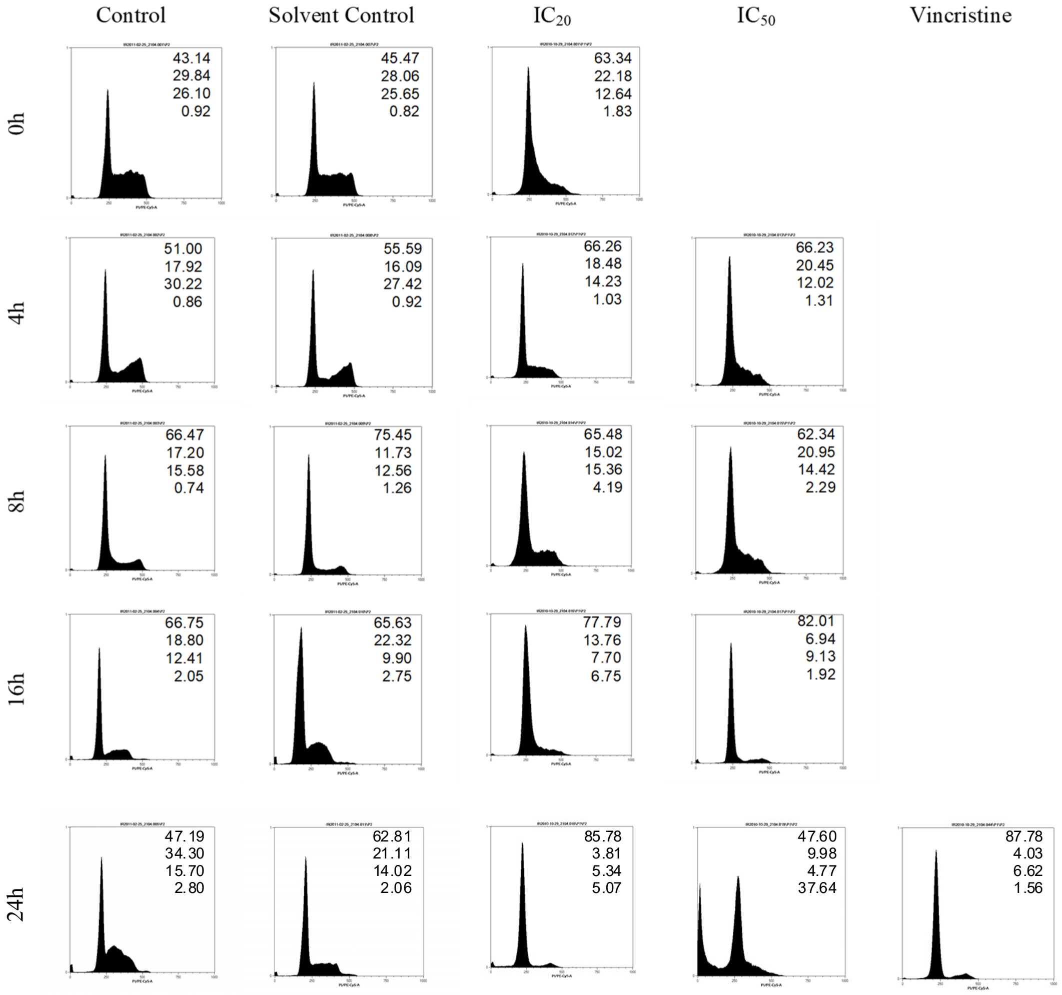

Figure S2. Effects of COX1 inhibitor on cell cycle status. B16F10 cells were treated with IC_{20} and IC_{50} concentration of Acetyl salicylic acid (ASA) inhibitor for 0, 4, 8, 16 and 24 hours. Flow cytometric analysis of DNA content was performed after propidium iodide staining. Percentage of cells in each phase of the cell cycle (sub-G_1, G_1, S, and G_2/M) is indicated. Representative data from three independent experiments are shown.

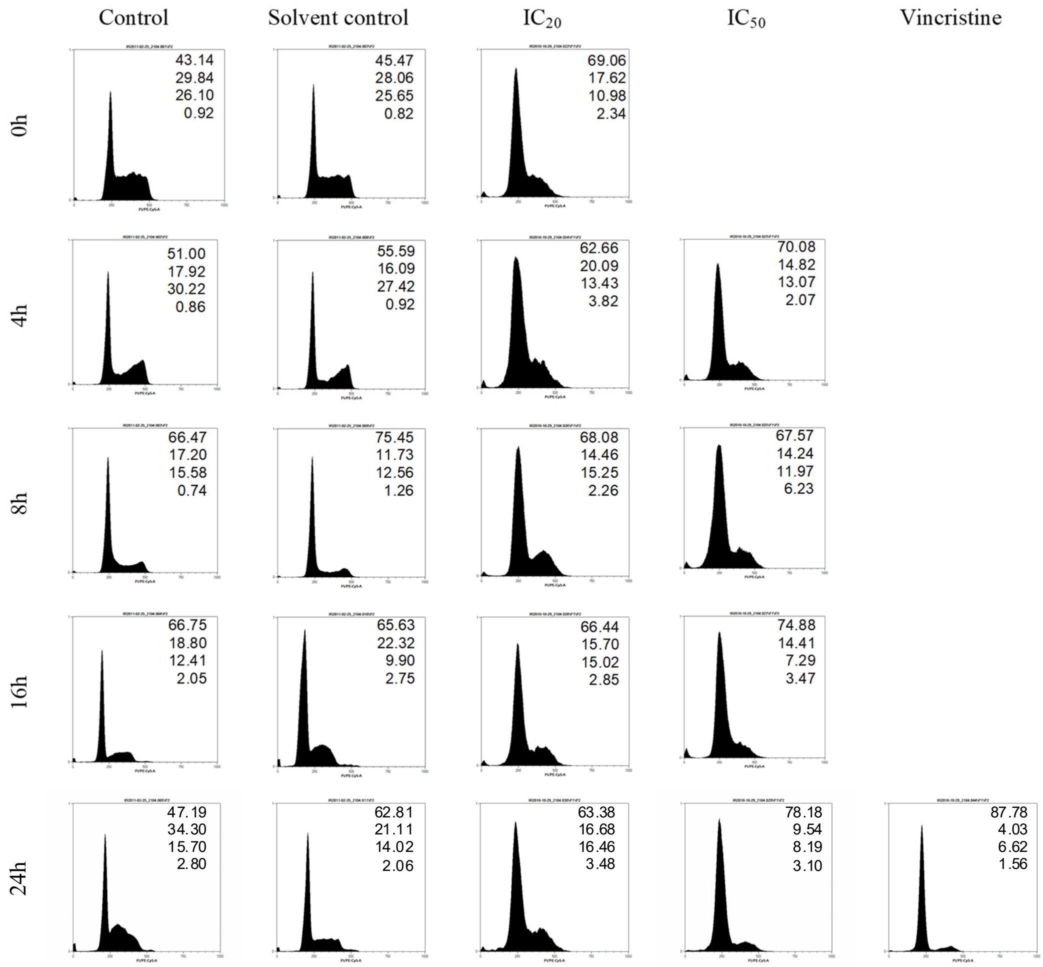

Figure S3. Effects of the 5-LOX inhibitor AA-861 on cell cycle status. B16F10 cells were treated with IC_{20} and IC_{50} concentration of AA-861 for 0, 4, 8, 16 and 24 hours. Flow cytometric analysis of DNA content was performed after propidium iodide staining. Percentage of cells in each phase of the cell cycle (sub-G_1, G_1, S, and G_2/M) is indicated. Representative data from three independent experiments are shown.

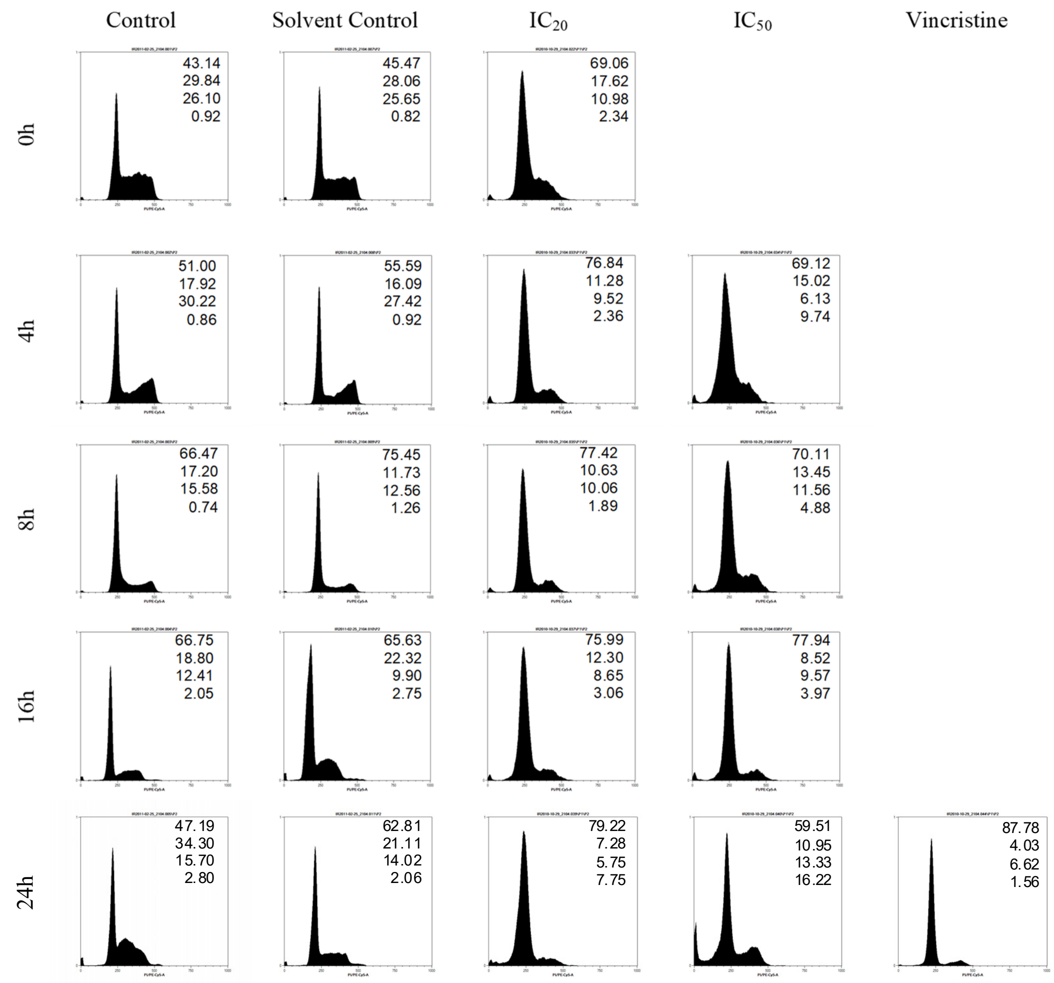

Figure S4. Effects of the FLAP inhibitor MK-886 on cell cycle status. B16F10 cells were treated with IC_{20} and µM IC_{50} concentration of MK-886 for 0, 4, 8, 16 and 24 hours. Flow cytometric analysis of DNA content was performed after propidium iodide staining. Percentage of cells in each phase of the cell cycle (sub-G_1, G_1, S, and G_2/M) is indicated. Representative data from three independent experiments are shown.

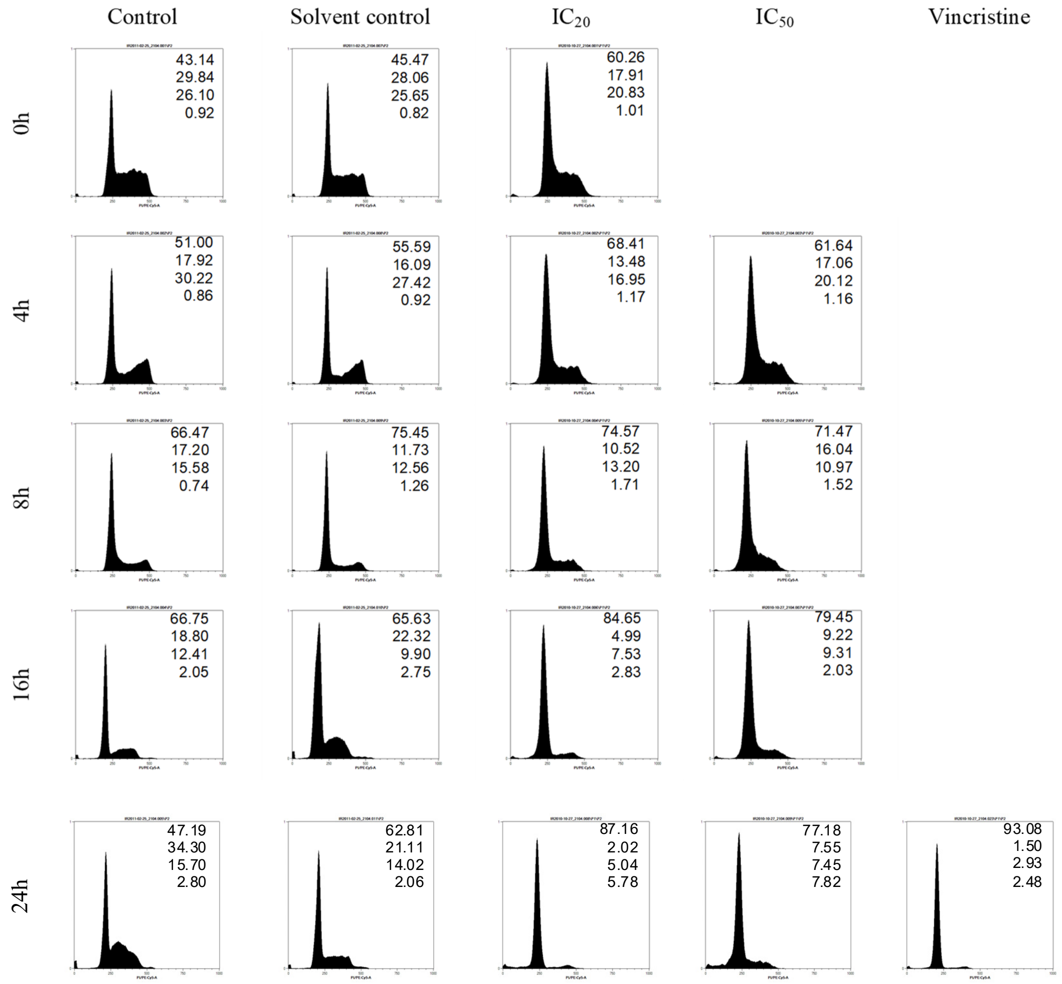

Figure S5. Effects of the 12-LOX inhibitor baicalein on cell cycle status. B16F10 cells were treated with IC_{20} and IC_{50} concentration of Baicalein for 0, 4, 8, 16 and 24 hours. Flow cytometric analysis of DNA content was performed after propidium iodide staining. Percentage of cells in each phase of the cell cycle (sub-G_1, G_1, S, and G_2/M) is indicated. Representative data from three independent experiments are shown.

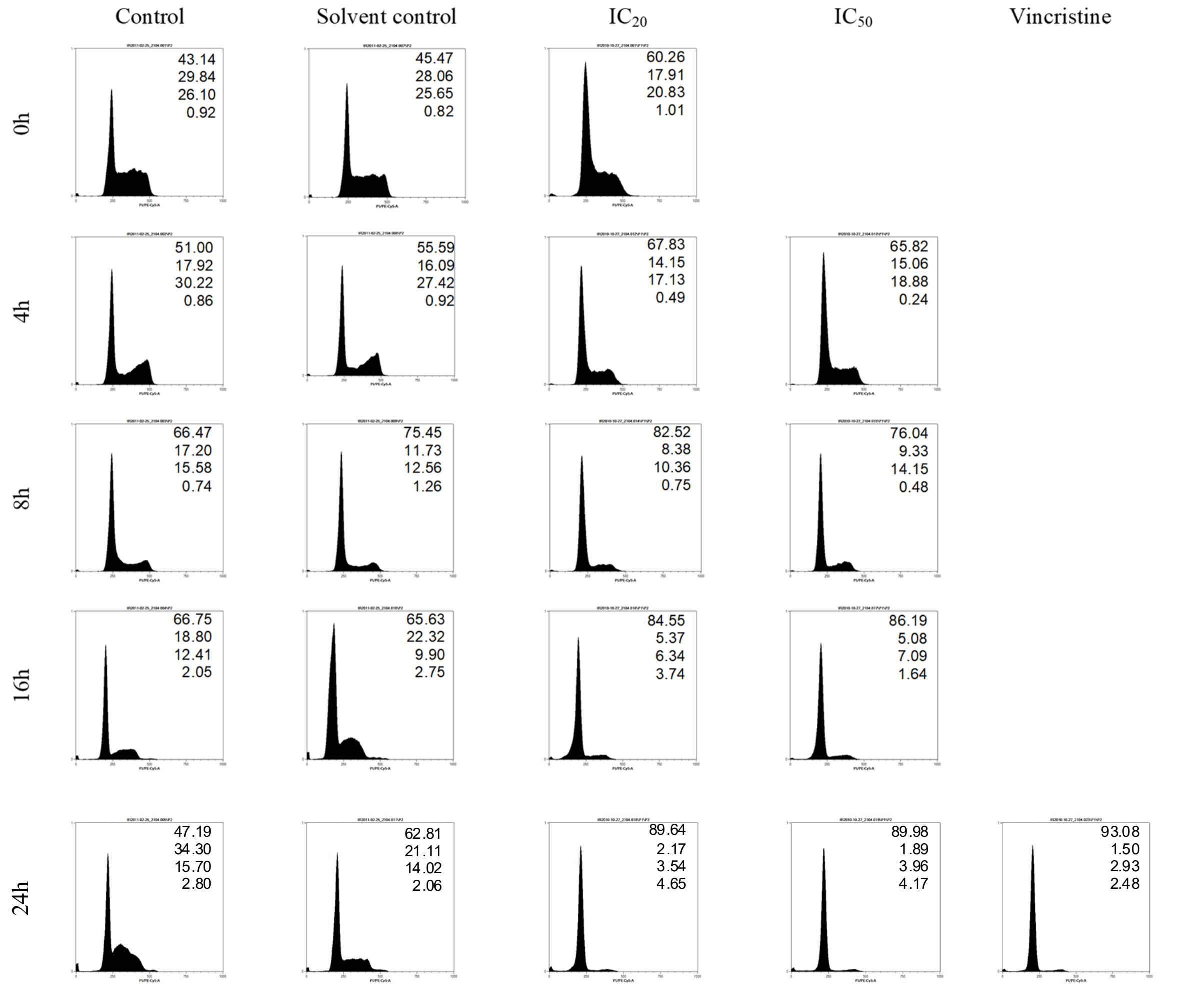

Figure S6. Effects of the 15-LOX inhibitor PD-146176 on cell cycle status. B16F10 cells were treated with IC_{20} and IC_{50} concentration of PD-146176 for 0, 4, 8, 16 and 24 hours. Flow cytometric analysis of DNA content was performed after propidium iodide staining. Percentage of cells in each phase of the cell cycle (sub-G_1, G_1, S, and G_2/M) is indicated. Representative data from three independent experiments are shown.

3. Numerical results from the combination values

Table S2. Combination index (CI) and fraction affected (f_a) values in B16F10 cells for DTIC or TMZ and meloxicam in combination based on the Calcusyn software.

DTIC (μM)	Meloxicam (μM)	f_a	CI	Effect	TMZ (μM)	Meloxicam (μM)	f_a	CI	Effect
**100**	0.31	0.643	0.011	Very strong synergism	**100**	0.31	0.136	0.255	Strong synergism
**100**	0.63	0.628	0.012	Very strong synergism	**100**	0.63	0.125	0.276	Strong synergism
**100**	3.13	0.644	0.012	Very strong synergism	**100**	3.13	0.159	0.295	Strong synergism
**100**	6.25	0.634	0.013	Very strong synergism	**100**	6.25	0.181	0.321	Synergism
**100**	31.25	0.637	0.020	Very strong synergism	**100**	31.25	0.260	0.461	Synergism
**100**	62.50	0.658	0.025	Very strong synergism	**100**	62.50	0.332	0.506	Synergism
**100**	312.50	0.832	0.018	Very strong synergism	**100**	312.50	0.317	2.097	Antagonism
**250**	0.31	0.362	0.064	Strong synergism	**250**	0.31	0.275	0.431	Synergism
**250**	0.63	0.358	0.065	Strong synergism	**250**	0.63	0.309	0.404	Synergism
**250**	3.13	0.361	0.070	Strong synergism	**250**	3.13	0.389	0.358	Synergism
**250**	6.25	0.379	0.072	Strong synergism	**250**	6.25	0.398	0.364	Synergism
**250**	31.25	0.489	0.097	Strong synergism	**250**	31.25	0.423	0.430	Synergism
**250**	62.50	0.409	0.104	Strong synergism	**250**	62.50	0.464	0.470	Synergism
**250**	312.50	0.685	0.083	Strong synergism	**250**	312.50	0.486	1.027	Moderate antagonism
**500**	0.31	0.136	0.330	Synergism	**500**	0.31	0.668	0.430	Synergism
**500**	0.63	0.129	0.354	Synergism	**500**	0.63	0.687	0.415	Synergism
**500**	3.13	0.155	0.345	Synergism	**500**	3.13	0.646	0.451	Synergism
**500**	6.25	0.168	0.364	Synergism	**500**	6.25	0.636	0.462	Synergism
**500**	31.25	0.161	0.802	Moderate synergism	**500**	31.25	0.603	0.521	Synergism
**500**	62.50	0.212	0.779	Moderate synergism	**500**	62.50	0.598	0.566	Synergism
**500**	312.50	0.485	0.365	Synergism	**500**	312.50	0.597	0.885	Slight synergism

Table S3. Combination index (CI), fraction affected (f_a) values and effect of combination therapy with ASA and DTIC or TMZ.

DTIC (μM)	ASA (μM)	f_a	CI	Effect	TMZ (μM)	ASA (μM)	f_a	CI	Effect
**100**	5	-	-	No effect	**100**	5	0.061	0.453	Synergism
**100**	10	-	-	No effect	**100**	10	0.051	0.706	Moderate synergism
**100**	50	-	-	No effect	**100**	50	0.070	0.993	Nearly additive
**100**	100	-	-	No effect	**100**	100	0.092	0.896	Slightly synergism
**100**	500	-	-	No effect	**100**	500	0.190	0.534	Synergism
**100**	1000	-	-	No effect	**100**	1000	0.313	0.271	Strong synergism
**100**	5000	0.380	0.996	Nearly additive	**100**	5000	0.289	0.924	Nearly additive
**250**	5	-	-	No effect	**250**	5	0.081	0.826	Slightly synergism
**250**	10	-	-	No effect	**250**	10	0.056	1.170	Moderate antagonism
**250**	50	-	-	No effect	**250**	50	0.121	0.785	Moderate synergism
**250**	100	-	-	No effect	**250**	100	0.132	0.830	Moderate synergism
**250**	500	0.012	1.266	Moderate antagonism	**250**	500	0.312	0.455	Synergism
**250**	1000	0.079	0.538	Synergism	**250**	1000	0.441	0.343	Synergism
**250**	5000	0.475	0.945	Nearly additive	**250**	5000	0.356	0.702	Moderate synergism
**500**	5	-	-	No effect	**500**	5	0.203	1.013	Nearly additive
**500**	10	-	-	No effect	**500**	10	0.191	1.049	Nearly additive
**500**	50		-	No effect	**500**	50	0.234	0.953	Nearly additive
**500**	100	-	-	No effect	**500**	100	0.254	0.920	Nearly additive
**500**	500	0.148	0.425	Synergism	**500**	500	0.406	0.691	Synergism
**500**	1000	0.249	0.406	Synergism	**500**	1000	0.482	0.608	Synergism
**500**	5000	0.528	0.940	Nearly additive	**500**	5000	0.479	0.682	Synergism

Table S4. Combination index (CI) and fraction affected (f_a) values in B16F10 cells for DTIC or TMZ and AA-861 in combination based on the Calcusyn software.

DTIC (μM)	AA-861 (μM)	f_a	CI	Effect	TMZ (μM)	AA-861 (μM)	f_a	CI	Effect
**100**	0.02	-	-	No effect	**100**	0.02	-	-	No effect
**100**	0.04	0.018	1.909	Antagonism	**100**	0.04	-	-	No effect
**100**	0.20	0.059	0.817	Moderate synergism	**100**	0.20	-	-	No effect
**100**	0.40	0.016	20.608	Very strong antagonism	**100**	0.40	0.028	4.529	Strong antagonism
**100**	2.00	0.021	58.668	Very strong antagonism	**100**	2.00	0.104	2.485	Antagonism
**100**	4.00	0.105	4.051	Strong antagonism	**100**	4.00	0.178	1.855	Antagonism
**100**	20.00	0.363	0.880	Moderate synergism	**100**	20.00	0.343	2.176	Antagonism
**250**	0.02	-	-	No effect	**250**	0.02	-	-	No effect
**250**	0.04	-	-	No effect	**250**	0.04	-	-	No effect
**250**	0.20	0.003	293.922	Very strong antagonism	**250**	0.20	-	-	No effect
**250**	0.40	0.008	83.245	Very strong antagonism	**250**	0.40	-	-	No effect
**250**	2.00	0.029	30.765	Very strong antagonism	**250**	2.00	0.054	7.744	Strong antagonism
**250**	4.00	0.102	4.439	Strong synergism	**250**	4.00	0.139	3.222	Antagonism
**250**	20.00	0.364	0.910	Nearly additive	**250**	20.00	0.302	3.136	Antagonism
**500**	0.02	0.119	0.376	Synergism	**500**	0.02	0.557	0.523	Synergism
**500**	0.04	0.159	0.297	Synergism	**500**	0.04	0.628	0.463	Synergism
**500**	0.20	0.162	0.351	Synergism	**500**	0.20	0.335	0.783	Moderate synergism
**500**	0.40	0.638	0.057	Very strong synergism	**500**	0.40	0.539	0.549	Synergism
**500**	2.00	0.638	0.064	Very strong synergism	**500**	2.00	0.501	0.645	Synergism
**500**	4.00	0.649	0.069	Very strong synergism	**500**	4.00	0.426	0.881	Slight synergism
**500**	20.00	0.662	0.124	Strong synergism	**500**	20.00	0.515	1.216	Moderate antagonism

Table S5. Combination index (CI) and fraction affected (f_a) values in B16F10 cells for DTIC or TMZ and MK-886 in combination based on the Calcusyn software.

DTIC (μM)	MK-886 (μM)	f_a	CI	Effect	TMZ (μM)	MK-886 (μM)	f_a	CI	Effect
**100**	0.015	-	-	No effect	**100**	0.015	-	-	No effect
**100**	0.030	-	-	No effect	**100**	0.030	-	-	No effect
**100**	0.150	0.029	3.271	Strong antagonism	**100**	0.150	-	-	No effect
**100**	0.300	0.045	2.879	Antagonism	**100**	0.300	0.009	48.036	Very strong antagonism
**100**	1.500	0.067	6.569	Strong antagonism	**100**	1.500	0.046	10.351	Very strong antagonism
**100**	3.0000	0.133	3.432	Strong antagonism	**100**	3.0000	0.174	1.432	Antagonism
**100**	15.000	0.505	0.604	Synergism	**100**	15.000	0.340	1.263	Moderate antagonism
**250**	0.015	-	-	No effect	**250**	0.015	-	-	No effect
**250**	0.030	-	-	No effect	**250**	0.030	-	-	No effect
**250**	0.150	0.036	2.518	Antagonism	**250**	0.150	-	-	No effect
**250**	0.300	0.034	5.048	Very strong antagonist	**250**	0.300	0.020	11.600	Very strong antagonist
**250**	1.500	0.050	11.551	Very strong antagonist	**250**	1.500	0.065	5.834	Strong antagonist
**250**	3.0000	0.068	12.835	Very strong antagonist	**250**	3.0000	0.163	1.973	Antagonist
**250**	15.000	0.409	1.222	Moderate antagonism	**250**	15.000	0.327	1.630	Antagonist
**500**	0.015	-	-	No effect	**500**	0.015	-	-	No effect
**500**	0.030	-	-	No effect	**500**	0.030	-	-	No effect
**500**	0.150	0.034	3.249	Antagonism	**500**	0.150	-	-	No effect
**500**	0.300	0.043	3.770	Strong antagonism	**500**	0.300	0.073	2.425	Antagonism
**500**	1.500	0.091	4.033	Strong antagonism	**500**	1.500	0.077	5.119	Strong antagonism
**500**	3.0000	0.074	11.213	Very strong antagonism	**500**	3.0000	0.161	2.584	Antagonism
**500**	15.000	0.289	3.157	Antagonism	**500**	15.000	0.292	2.524	Antagonism

Table S6. Combination index (CI) and fraction affected (f_a) values in B16F10 cells for DTIC or TMZ and Baicalein in combination based on the Calcusyn software.

DTIC (μM)	Baicalein (μM)	f_a	CI	Effect	TMZ (μM)	Baicalein (μM)	f_a	CI	Effect
**100**	0.025	-	-	No effect	**100**	0.025	-	-	No effect
**100**	0.050	-	-	No effect	**100**	0.050	-	-	No effect
**100**	0.250	-	-	No effect	**100**	0.250	-	-	No effect
**100**	0.500	-	-	No effect	**100**	0.500	0.01	18.264	Very strong antagonism
**100**	2.500	-	-	No effect	**100**	2.500	0.034	9.992	Strong antagonism
**100**	5.000	0.017	5.136	Strong antagonism	**100**	5.00	0.079	4.238	Strong antagonism
**100**	25.000	0.301	1.917	Antagonism	**100**	25.00	0.299	1.300	Moderate antagonism
**250**	0.025	-	-	No effect	**250**	0.025	-	-	No effect
**250**	0.050	-	-	No effect	**250**	0.050	-	-	No effect
**250**	0.250	-	-	No effect	**250**	0.250	-	-	No effect
**250**	0.500	-	-	No effect	**250**	0.500	0.003	153.422	Very strong antagonism
**250**	2.500	-	-	No effect	**250**	2.500	0.031	12.486	Very strong antagonism
**250**	5.000	0.074	1.697	Antagonism	**250**	5.000	0.098	3.295	Antagonism
**250**	25.000	0.359	1.596	Antagonism	**250**	25.000	0.362	1.047	Slight antagonism
**500**	0.025	0.099	0.425	Synergism	**500**	0.025	-	-	No effect
**500**	0.050	0.113	0.386	Synergism	**500**	0.050	-	-	No effect
**500**	0.250	0.104	0.456	Synergism	**500**	0.250	-	-	No effect
**500**	0.500	0.115	0.469	Synergism	**500**	0.500	0.036	3.950	Strong antagonism
**500**	2.500	0.088	1.078	Nearly additive	**500**	2.500	0.131	1.975	Antagonism
**500**	5.000	0.126	1.241	Moderate antagonism	**500**	5.000	0.238	1.324	Moderate antagonism
**500**	25.000	0.596	0.774	Nearly additive	**500**	25.000	0.384	1.277	Moderate antagonism

Table S7. Combination index (CI) and fraction affected (f_a) values in B16F10 cells for DTIC or TMZ and PD-146176 in combination based on the Calcusyn software.

DTIC (μM)	PD-146176 (μM)	f_a	CI	Effect	TMZ (μM)	PD-146176 (μM)	f_a	CI	Effect
**100**	0.005	-	-	No effect	**100**	0.005	-	-	No effect
**100**	0.010	-	-	No effect	**100**	0.010	-	-	No effect
**100**	0.050	-	-	No effect	**100**	0.050	-	-	No effect
**100**	0.100	-	-	No effect	**100**	0.100	-	-	No effect
**100**	0.500	-	-	No effect	**100**	0.500	0.045	2.318	Antagonism
**100**	1.000	0.027	1.255	Moderate antagonism	**100**	1.000	0.149	1.246	Moderate antagonism
**100**	5.000	0.206	1.590	Moderate antagonism	**100**	5.000	0.358	1.707	Antagonism
**250**	0.005	-	-	No effect	**250**	0.005	-	-	No effect
**250**	0.010	-	-	No effect	**250**	0.010	-	-	No effect
**250**	0.050	-	-	No effect	**250**	0.050	-	-	No effect
**250**	0.100	-	-	No effect	**250**	0.100	-	-	No effect
**250**	0.500	-	-	No effect	**250**	0.500	0.084	1.744	Antagonism
**250**	1.000	0.041	1.232	Moderate antagonism	**250**	1.000	0.132	1.791	Antagonism
**250**	5.000	0.168	1.902	Antagonism	**250**	5.000	0.339	2.077	Antagonism
**500**	0.005	-	-	No effect	**500**	0.005	-	-	No effect
**500**	0.010	-	-	No effect	**500**	0.010	-	-	No effect
**500**	0.050	-	-	No effect	**500**	0.050	-	-	No effect
**500**	0.100	-	-	No effect	**500**	0.100	-	-	No effect
**500**	0.500	-	-	No effect	**500**	0.500	0.082	2.557	Antagonism
**500**	1.000	0.031	1.987	Antagonism	**500**	1.000	0.097	3.101	Antagonism
**500**	5.000	0.192	1.858	Antagonism	**500**	5.000	0.322	2.618	Antagonisms
